# The association between genetically predicted systemic inflammatory regulators and endometriosis: A bidirectional Mendelian randomization study

**DOI:** 10.1097/MD.0000000000038972

**Published:** 2024-07-19

**Authors:** Yufeng Liu, Yuhong Liu, Wangshu Li, Xiaoxia Sun

**Affiliations:** a The First Affiliated Hospital of Xi’an JiaoTong University Yulin Hospital, Shanxi Province, China; b Dalian Women and Children’s Medical Group, Dalian, Liaoning Province, China.

**Keywords:** cytokines, endometriosis, inflammation, Mendelian randomization

## Abstract

Elevated levels of various cellular inflammatory markers have been observed in patients with endometriosis (EMs). However, a causal relationship between these markers and EMS has not been firmly established. This study aimed to assess the causality between cellular inflammatory markers and the onset of EMS using a bidirectional Mendelian randomization approach. Genetic associations for EMs were derived from the largest and most recent genome-wide association study (GWAS) involving 1937 EMS cases and 245,603 controls of European ancestry. Single nucleotide polymorphisms associated with 41 cellular cytokines and other systemic inflammatory regulators were identified from 8293 Finnish participants. Estimates were obtained using inverse-variance weighted, with sensitivity analyses conducted using MR-Egger, weighted median, and MR-PRESSO. Among the 41 systemic inflammatory regulators included in the analysis, none were associated with the risk of EMs. Elevated levels of IL-6 were associated with an increased risk of EMs (OR = 1.351, 95%CI = 1.015–1.797). Conversely, genetically predicted elevated levels of platelet-derived growth factor (PDGF-BB) were associated with a reduced risk of EMs (OR = 0.856, 95%CI = 0.742–0.987). Genetically predicted elevations in IL-6 may contribute to an increased risk of EMs, while elevated PDGF-BB levels appear protective, suggesting potential therapeutic targets for EMs. Other systemic inflammatory regulators seem unrelated to EMs risk, potentially representing downstream effects or consequences of shared factors between inflammation and EMs.

## 1. Introduction

Endometriosis (EMs) is characterized as a benign, proliferative, chronic ailment, principally distinguished by the ectopic presence and growth of functional endometrial tissue, glands, and stroma outside the uterine cavity.^[1]^ Recognized as one of the predominant gynecological disorders, its clinical manifestations predominantly encompass menstrual irregularities, chronic pelvic discomfort, dysmenorrhea, and infertility.^[2]^ Approximately 10% of women of reproductive age are afflicted with EMs, with the prevalence soaring to an alarming 50% among infertile women.^[3]^ Furthermore, endometriosis is perceived as a debilitating condition, potentially inflicting severe detriment to individual social interactions, sexual well-being, and psychological health.^[2–4]^ The etiological underpinnings of EMs remain elusive; however, postulated pathophysiological mechanisms encompass retrograde menstruation, a persistent inflammatory milieu, and intracavitary metastasis.^[5,6]^ While the pathophysiology of endometriosis remains incompletely elucidated, mounting evidence suggests an intricate interplay of hormonal, immunological, genetic, and environmental factors in its genesis and progression.^[7,8]^ Notably, numerous studies have underscored aberrant immune and inflammatory responses in EMs, particularly elevated levels of pro-inflammatory cytokines, including tumor necrosis factor-alpha (TNF-α), interleukin (IL)-1β, IL-6, and IL-17A.^[9–12]^ However, the elevation of these cytokines remains a topic of debate: are they intrinsically involved in the pathogenesis of EMs, or are they merely downstream effects subsequent to the onset of EMs?^[13,14]^

Mendelian randomization (MR) is an emergent bioinformatics technique that employs single nucleotide polymorphisms (SNPs) associated with risk factors as instrumental variables (IVs) to ascertain causal relationships between risk factors and specific diseases.^[15,16]^ Given that genetic variations in mammals are established at the zygote stage and remain unchanged throughout life, MR studies can circumvent potential biases from confounders or other interferences.^[17]^

In this study, we employed 2-sample MR to investigate the potential influence of certain systemic inflammatory factors in the pathogenic mechanisms of EMs. Additionally, this research assessed the association between genetically predicted EMs and systemic cytokines, aiming to elucidate the genuine causal relationship between EMs and these inflammatory cytokines.

## 2. Materials and methods

### 2.1. Data sources

SNPs related to systemic inflammatory regulators were obtained and selected from the summary statistics of the largest and most recent genome-wide association study (GWAS) involving 41 systemic inflammatory regulators. This study included a total of 8293 Finnish participants from 3 different cohort studies: the Cardiovascular Risk in Young Finns Study, the FINRISK1997 study, and the FINRISK200225 study.^[18]^ In the analysis, genetic associations were adjusted for age, sex, body mass index, and the first 10 genetic principal components. For the investigation of genetic associations with endometriosis in individuals of European ancestry, a GWAS meta-analysis was conducted. The analysis included a total of 1937 endometriosis cases and 245603 controls of European descent (GCST90044411). In this analysis, the genetic associations were adjusted for age within the cohort.

### 2.2. The selection of genetic instruments

For each of the 41 systemic inflammatory regulators (Supplementary Table S1) http://links.lww.com/MD/N239, we extracted the SNPs that significantly predicted exposures at the genome-wide level of significance (*P* < 5 × 10^–6^). To avoid potential pleiotropy, we removed SNPs that were associated with more than one systemic inflammatory regulator. We checked for linkage disequilibrium (LD) using the European 1000 G reference panel and selected SNPs with r^2^ < 0.01 to account for the effect of correlated SNPs. To ensure the validity of instrumental variables (IVs), we calculated the average SNP-specific F-statistics and considered IVs with F-statistics > 10 as strong IVs for MR analysis. To investigate the causal effect of endometriosis on systemic inflammatory regulators, we used a genome-wide level of significance (*P* < 5 × 10^-6^) to select SNPs (Supplementary Table S2) http://links.lww.com/MD/N239. The selection procedures for these SNPs were the same as those used for the systemic inflammatory regulators.

### 2.3. Statistical analysis

Inverse-variance weighted (IVW) method was used as the primary analysis for MR. Additionally, 3 other methods were employed as references: the weighted median estimator (WME), MR-Egger regression, simple mode, and weighted mode. Sensitivity analyses included tests for heterogeneity and pleiotropy. The significance of any pleiotropy was validated by the MR-Egger intercept test.^[19]^ Cochran Q test was employed for heterogeneity assessment, and *P* > .05 indicated no heterogeneity.^[20]^ Furthermore, we applied the “leave one out of analysis” method to detect any significant association dominated by a single SNP. Effect estimates for binary outcomes were represented as odds ratios (ORs) with 95% confidence intervals.

We estimated the strength of the IVs using the F statistic; where F < 10, weak instrumental variable bias was assumed. The F statistic {calculated as F = [(n − k − 1)/ k] * [R^2^/ (1 − R^2^)]; R^2^ = 2* EAF * (1 − EAF) * beta2, and indicating the proportion of variance in the exposure explained by the genetic variants; EAF = effect allele frequency; n = sample size; and k = number of IVs) indicates the strength of the relationship between the IVs and exposure. After removing the aforementioned non-compliant instrumental variables, we repeated the MR analysis to obtain the final MR estimate. Without heterogeneity and pleiotropy, IVW is a more reliable fitting model.^[21]^ For binary outcomes, the effect estimates are represented as ORs with a 95% confidence interval.

Moreover, we performed bidirectional MR analysis to test the association between genetically predicted EMs and systemic inflammatory regulators. MR analyses and sensitivity analyses were performed in R (version 4.0.2) using the TwoSampleMR package (version 0.5.5). For laboratory data, continuous variables were expressed as mean ± standard deviation (SD) and Student *t* test was adopted for the comparison between the 2 groups. Binary logistic regression analyses were used to determine the ORs and the corresponding 95% CIs. For all comparisons, a two-sided *P* value < .05 was considered statistically significant and statistical analyses were performed using SPSS version 22.0.

### 2.4. Ethical approval

The GWAS data utilized in this study are publicly available de-identified datasets. The Institutional Review Board (IRB) has approved these data; therefore, no additional ethical approval is required.

## 3. Results

### 3.1. Instrumental variable selection

**Figure 1.**
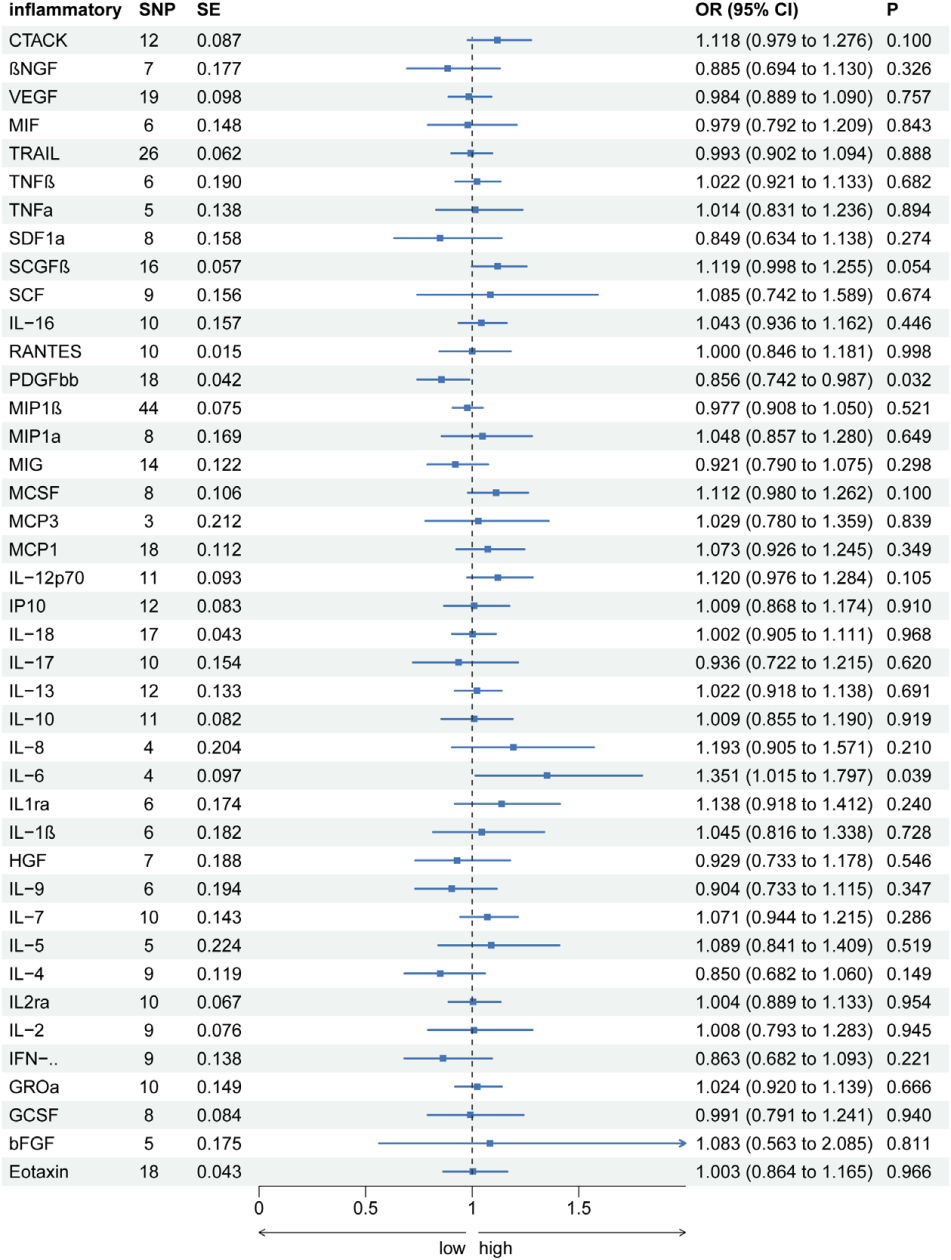
The association of systemic inflammatory regulators (exposure) with EMs (outcome) using SNPs reaching the *P* < 5 × 10^–6^ significance level: the inverse-variance weighted (IVW) method was applied as the primary method for MR analysis.

For the 41 cytokines, we identified at least 2 genetic variants available at *P* < 5 × 10^–6^ and LD (r^2^ < 0.1, distance ≥ 500 kb). The numbers of IVs for each cytokine are shown in Figure 1. The associations of IVs with each circulating cytokine level can be found in Supplementary Table S3 http://links.lww.com/MD/N239. All F-statistics were above 10, indicating that the results were less likely to be affected by the weak instrument bias.

### 3.2. Causal effects of cytokine levels on the risk of endometriosis

Among the 41 cytokines examined, genetically predicted platelet-derived growth factor-BB (PDGF-bb) and IL-6 showed a significant association with the risk of endometriosis in at least 1 MR method. Specifically, PDGF-bb demonstrated a protective role against endometriosis: an increase of 1 SD in PDGF-bb levels was associated with a decreased OR of 0.856 (95% CI: 0.742–0.987, *P* = .032). Conversely, elevated levels of IL-6 were linked to a heightened risk of endometriosis, yielding an OR of 1.351 (95% CI: 1.015–1.797, *P* = .039). However, the MR-Egger and Weighted median methods did not demonstrate significant associations for IL-6 (MR-Egger: OR: 1.118, 95% CI: 0.789–1.767, *P* = .503; Weighted median: OR: 1.267, 95% CI: 0.931–1.724, *P* = .132). Our analyses did not identify significant associations between other cytokines and endometriosis. Sensitivity analyses of all MR methods revealed no evidence of pleiotropy or heterogeneity (Fig. 1 and Supplementary Table S3) http://links.lww.com/MD/N239.

### 3.3. Causal impact of endometriosis on circulating cytokine levels

**Figure 2.**
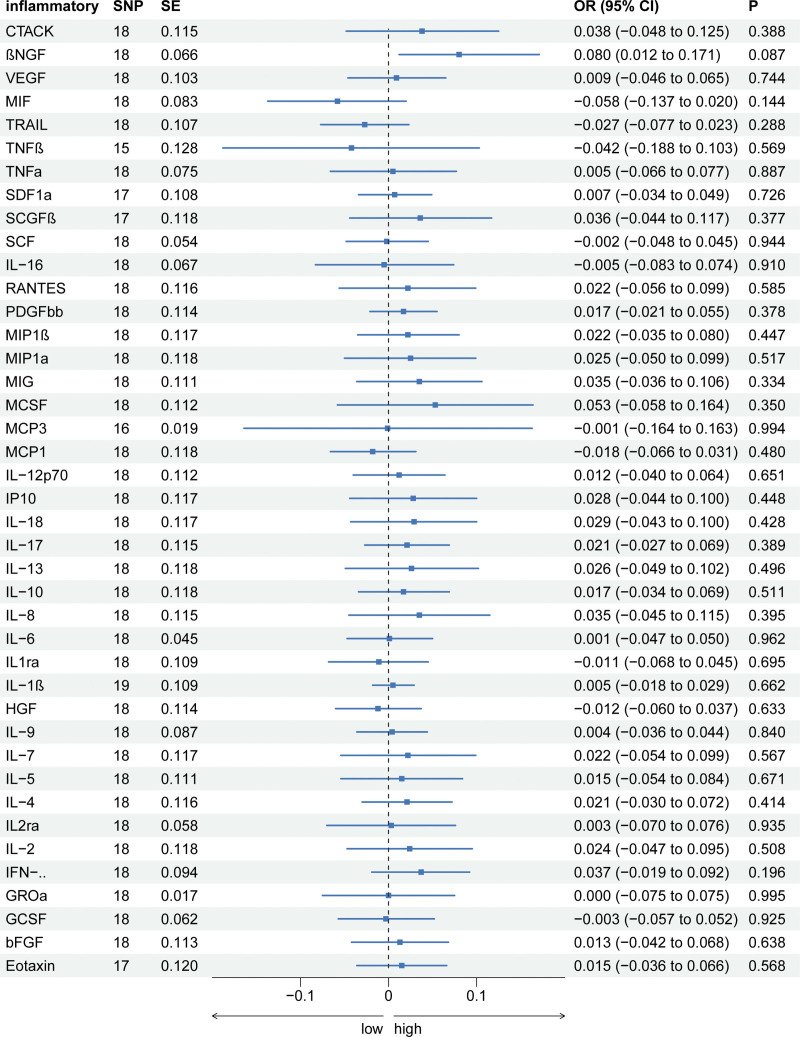
The association of EMs (Exposure) with systemic inflammatory regulators (outcome) using Mendelian randomization with SNPs reaching the *P* < 5 × 10^–6^ significance level: the inverse-variance weighted (IVW) method was applied as the primary method for MR analysis.

Our investigation also considered the potential causal relationships of endometriosis on circulating cytokine levels. However, our results did not indicate significant associations between endometriosis and any systemic inflammatory modulators across the MR methods utilized. Furthermore, no evidence of horizontal pleiotropy or heterogeneity was detected in our analyses (Fig. 2 and Supplementary Table S4) http://links.lww.com/MD/N239.

## 4. Discussion

In this comprehensive study, we employed a 2-sample MR approach, leveraging the most expansive GWAS dataset currently available, encompassing data from 8293 individuals, to elucidate the putative causal interplay between systemic cytokines and EMs. Genetic correlations were meticulously adjusted for age, gender, body mass index, and the inaugural 10 principal genetic components. Intriguingly, our results highlight an increased risk of EMs concomitant with elevated IL-6 levels (OR = 1.351, 95% CI = 1.015–1.797). In stark contrast, a genetically predicted augmentation in Platelet-derived growth factor BB (PDGF-BB) was inversely associated with EMs prevalence (OR = 0.856, 95% CI = 0.742–0.987). Of the 39 cytokines probed, none manifested a discernible causal linkage with EMs (*P* > .05). To the best of our knowledge, this endeavor marks the pioneering, in-depth MR exploration into the causal nexus between genetically inferred systemic inflammation and EMs. These revelations provide groundbreaking insights, demystifying the intricate nexus between EMs and inflammatory mediators, holding potential therapeutic ramifications.

### 4.1. Analysis and implications

Upon evaluating the instrumental variables (IVs) included in our study, we discerned that elevated levels of IL-6 significantly augment the risk of EMs onset (OR = 1.351, 95% CI = 1.015–1.797). Using the inverse-variance weighted (IVW) method, we observed a significant association (*P* < .05). Although other methods yielded *P* > .05, the directionality of the beta was consistent, bolstering the reliability of the IVW results. Subsequent tests for pleiotropy and heterogeneity yielded *P* > .05, underscoring the robustness and validity of our findings.

IL-6 serves as a hallmark of acute inflammation, possessing myriad biological functions, including the induction of vascular endothelial growth factor expression and the differentiation and activation of lymphocytes, notably T and B cells.^[18,22]^ Furthermore, IL-6 plays a pivotal role in the reproductive system, modulating folliculogenesis, ovarian steroidogenesis, and interactions with oocyte implantation.^[23]^ While numerous studies have reported elevated concentrations of IL-6 in the peritoneal fluid and blood of endometriosis patients, the data remains somewhat heterogeneous.^[24,25]^ Nonetheless, the utility of IL-6 as a biomarker for EMs, aiding in diagnosis, subtype differentiation, and prognostication, has garnered consensus in the medical community.^[26]^ Our study reaffirms the positive correlation between elevated IL-6 levels and EMs pathogenesis.

Some researchers posit that IL-6 can induce NOTCH1 expression,^[27]^ promoting endometriotic lesions through E-cadherin mediation in ectopic glandular epithelial cells, potentially elucidating the pathophysiological mechanism by which IL-6 exacerbates EMs. Clarifying the relationship between IL-6 and EMs pathogenesis will undoubtedly enhance our understanding of EMs. IL-6, beyond serving as a disease marker, emerges as a potential therapeutic target for EMs intervention. Notably, some scholars have experimented with tocilizumab (an IL-6 inhibitor) in animal models for EMs treatment, observing a marked reduction in endometriotic lesion volume and atrophy of ectopic endometrial-like epithelium, further substantiating the therapeutic potential of targeting IL-6 in EMs.^[28]^

### 4.2. Platelet-derived growth factor and endometriosis: A novel perspective

Intriguingly, genetically predicted elevations in platelet-derived growth factor (PDGF-BB) were associated with a reduced risk of endometriosis (OR = 0.856, 95% CI = 0.742–0.987). Through multiple statistical methodologies, we consistently observed that increased levels of PDGF-BB significantly attenuate the incidence of EMs. PDGF is a platelet-derived angiogenic factor, and the pivotal role of angiogenesis and angiogenic factors in the pathophysiology of endometriosis has been well-established.^[29,30]^

Compared to widely studied cytokines such as IL-6 and IL-17, research on PDGF-BB in the context of EMs remains relatively sparse. A small-scale randomized controlled trial reported no discernible difference in PDGF mRNA expression between endometriosis patients and healthy controls.^[31]^ Another study indicated that a decline in PDGF in endometriosis patients was only evident during the late secretory phase of the endometrium, with no differences observed in other phases compared to healthy individuals.^[32]^

It imperative to note that PDGF not only promotes angiogenesis but also plays a dual role in balancing pro-angiogenic and anti-angiogenic activities.^[33]^ In healthy individuals, maintaining optimal levels of PDGF-BB may support the healthy proliferation of endometrial cells. Conversely, diminished PDGF-BB levels might compromise the body regulatory mechanisms over vasculature and endometrial cells, potentially precipitating endometriosis. Thus, sustaining certain PDGF expression levels might mitigate the onset of EMs. However, excessively elevated PDGF levels might also predispose individuals to EMs. These hypotheses warrant further experimental validation.

### 4.3. Cytokines and endometriosis: Challenging established paradigms

On another note, our investigation revealed that several cytokines, including Interleukin-17, Interleukin-18, Cutaneous T-cell attracting chemokine, TNF-related apoptosis-inducing ligand, and tumor necrosis factor-alpha, did not exhibit a direct causal relationship with the onset of endometriosis (*P* > .05). This finding stands in contrast to numerous studies that have posited a robust association between endometriosis and inflammatory markers.^[34–37]^ Our results challenge the prevailing notion of a direct causal link between the 2.

Given that inflammation is a primary driver in the pathogenesis of endometriosis and cytokines are products of the inflammatory process, it plausible that clinical studies discern an association between cytokines and endometriosis. However, the inflammatory markers themselves might not have a direct causal relationship with the onset of endometriosis. Instead, they could be downstream effects post-EMs onset or results of common factors leading to both inflammation and endometriosis. This intriguing perspective necessitates further exploration.

### 4.4. MR and its implications in studying inflammatory cytokines and endometriosis

A significant strength of our study lies in the utilization of the Mendelian Randomization (MR) approach to investigate the association between cellular inflammatory markers and endometriosis (EMs). Consequently, our findings are less likely to be influenced by reverse causation and potential confounding factors. Furthermore, we harnessed the largest GWAS dataset to date, extracting SNPs related to systemic inflammatory modulators and EMs.

However, our study is not without limitations. Firstly, the GWAS data for these cytokines did not provide effect allele frequencies (EAF), preventing us from identifying palindromic SNPs to ascertain alignment in the same direction for exposure and outcome. To address this challenge, we employed MR-Egger and MR-PRESSO analyses to test for horizontal pleiotropy, but this does not fully mitigate the limitation. Another concern is that our MR study is exclusively based on European ancestry, leaving questions about the applicability of our findings to other ethnicities or regions. Further research focusing on the association between systemic inflammatory modulators, cytokines, and EMs is warranted.

In conclusion, employing a 2-sample MR approach, our evidence suggests that genetically predicted elevated levels of IL-6 are associated with an increased risk of EMs, while elevated levels of platelet-derived growth factor are linked to a reduced incidence of EMs. For the other 39 cellular inflammatory markers, our MR study did not find evidence supporting a potential causal relationship with EMs risk. Further exploration of upstream factors and other systemic inflammatory cytokines in relation to EMs will provide valuable insights into the etiology and therapeutic avenues for EMs.

## Author contributions

**Data curation:** Yuhong Liu, Wangshu Li.

**Writing – original draft:** Yufeng Liu.

**Writing – review & editing:** Xiaoxia Sun.

## Supplementary Material


