# Supplementary material for: The association between genetically predicted systemic inflammatory regulators and endometriosis: A bidirectional Mendelian randomization study
Source: Medicine (Baltimore). 2024 Jul 19;103(29):e38972. doi: 10.1097/MD.0000000000038972 (PMC11398771; doi:10.1097/MD.0000000000038972)
Supplement: Supplementary file 1 [file medi-103-e38972-s001.docx]

**Supplementary Table S1. Characteristics of the genetic instrument variables for the systemic inflammatory regulators in the Mendelian randomization study at the genome-wide significance level (P < 5 × 10^–6^).**

| **Systematic inflammatory regulators** | **Number of SNPs** | **SNP** | **chr** | **pos** | **Effect allele** | **Other allele** | **Beta of exposure** | **SE of exposure** | **P of exposure** | **Beta of outcome** | **SE of outcome** | **P of outcome** | **F statistics** |
| --- | --- | --- | --- | --- | --- | --- | --- | --- | --- | --- | --- | --- | --- |
| CTACK | 12 | rs116303454  rs12438356  rs141420735  rs145902143  rs2070074  rs2731674  rs3766110  rs55764737  rs57338032  rs58704839  rs7333764  rs76395525 | 3  15  9  12  9  5  1  15  15  9  13  15 | 27294655  69579577  34515043  20944907  34649442  176839890  169515183  61323414  78798939  34680554  34208801  79741391 | G  C  C  G  G  G  C  C  G  G  C  G | A  T  T  A  A  T  A  T  A  A  T  A | -0.383  -0.1548  0.3966  0.2838  -0.4467  0.1333  0.1287  -0.5313  -0.1583  -0.1785  -0.2773  -0.5277 | 0.0816  0.0337  0.0789  0.0581  0.0374  0.0267  0.0278  0.0972  0.0317  0.0284  0.0593  0.1083 | 3.27E-06  4.46E-06  5.82E-07  1.03E-06  1.79E-32  5.64E-07  3.86E-06  4.62E-08  6.23E-07  3.29E-10  2.85E-06  9.55E-07 | 0.0742993  -0.0324981  0.156302  0.100741  0.0225434  0.021637  0.000882141  -0.143529  -0.0205743  -0.035787  -0.183969  0.00252274 | 0.111207564  0.051651236  0.186542987  0.076703631  0.055113993  0.037269661  0.038405719  0.090077827  0.044220115  0.037117229  0.118989892  0.144864946 | 0.504061  0.529229  0.402094  0.189055  0.682516  0.561542  0.981675  0.111073  0.641738  0.334965  0.122083  0.986106 | 22.03013865  21.09998327  25.26686811  23.86011417  142.6555592  24.92514974  21.43223694  29.87769585  24.93694832  39.50388068  21.86705778  23.7419986 |
| βNGF | 7 | rs28637706  rs67476890  rs71641308  rs72780728  rs73472576  rs7970581  rs9436119 | 19  15  1  10  18  12  1 | 34285368  62791494  78086718  17603701  72124182  113265248  150467753 | G  C  C  G  C  G  G | T  T  T  A  T  T  A | 0.1589  -0.1769  -0.2043  -0.1883  0.1181  -0.138  0.1121 | 0.0263  0.0379  0.0432  0.0403  0.0252  0.0282  0.0246 | 1.42E-09  3.13E-06  2.30E-06  2.99E-06  2.69E-06  9.27E-07  3.91E-06 | -0.0288373  0.0245846  -0.0966493  0.00109773  -0.0883773  0.00678217  -0.00654343 | 0.03355658  0.0497775  0.064185097  0.057160161  0.0342628  0.038371607  0.033155903 | 0.39014  0.621384  0.132121  0.984678  0.0098974  0.859705  0.843551 | 36.50365048  21.78598729  22.36501736  21.83185045  21.96335664  23.94748755  20.76543393 |
| VEGF | 19 | rs10153304  rs10934631  rs114773511  rs143479231  rs3025021  rs34881325  rs4082730  rs41282660  rs4507572  rs60987108  rs62401205  rs67798973  rs6920532  rs6921438  rs73418461  rs73736926  rs74675876  rs75967707  rs8045833 | 17  3  6  3  6  9  15  6  6  6  6  6  6  6  10  6  6  6  16 | 7721931  122697600  43796181  193110794  43749163  2622134  90523558  44197006  44135095  44427217  43855489  43882777  43793430  43925607  120222996  44006091  43963995  43130066  88575439 | G  C  C  G  C  C  G  G  C  G  C  G  C  G  G  G  C  C  G | A  T  T  A  T  T  A  A  T  A  A  A  T  A  A  T  T  T  A | -0.1547  0.1151  -0.2186  0.2598  -0.0985  0.1082  -0.2522  0.1613  -0.1007  -0.1852  0.2039  -0.1389  -0.1803  0.49  0.2492  0.1899  0.2822  -0.1792  -0.108 | 0.0325  0.0245  0.0442  0.0491  0.0203  0.0189  0.0534  0.0263  0.0171  0.0392  0.0412  0.0175  0.0267  0.0175  0.0521  0.036  0.0366  0.0391  0.0211 | 1.94E-06  2.47E-06  1.25E-06  1.90E-07  1.40E-06  1.04E-08  2.64E-06  1.33E-09  3.34E-09  2.95E-06  1.77E-06  1.29E-15  8.68E-12  2.09E-171  1.68E-06  1.82E-07  7.62E-15  2.12E-06  2.83E-07 | -0.00401539  -0.062499  0.00075878  -0.0370284  0.0221488  0.0419387  0.00631584  -0.00631204  0.0309263  -0.03338  0.0382674  0.0620488  0.0348873  0.020915  0.0125675  -0.0629285  -0.044745  -0.0454745  0.0171807 | 0.053351929  0.040595404  0.099085586  0.146273355  0.036546291  0.035441981  0.090982904  0.050393682  0.032678739  0.075665777  0.089028779  0.033611764  0.057524801  0.032668412  0.072143937  0.082253962  0.106352164  0.068881593  0.036662735 | 0.940006  0.123668  0.99389  0.800156  0.544483  0.236688  0.944657  0.900322  0.343958  0.659104  0.667318  0.0648858  0.5442  0.522029  0.861708  0.444241  0.673956  0.509136  0.639345 | 22.6576  22.07082049  24.45996192  27.99724574  23.54400738  32.77411047  22.30527851  37.61466842  34.67901235  22.32080383  24.49288929  62.99823673  45.60042924  784  22.87813558  27.825625  59.44999851  21.00499081  26.19887244 |
| MIF | 6 | rs113218956  rs118055855  rs12594190  rs13142904  rs141009259  rs78098071 | 22  11  15  4  2  5 | 25224834  29888572  25036455  54318414  207976283  163309739 | G  C  G  C  C  C | A  T  A  T  T  T | 0.8948  -0.6907  -0.1355  0.223  0.6178  0.4867 | 0.1879  0.15  0.0267  0.0425  0.1322  0.0918 | 2.26E-06  4.13E-06  3.70E-07  2.56E-07  2.47E-06  1.78E-07 | -0.131931  -0.096005  0.0127082  -0.0228623  0.0186753  -0.104228 | 0.267772557  0.132493951  0.038888486  0.066322672  0.163518424  0.155643689 | 0.622226  0.468698  0.74383  0.73031  0.909072  0.503076 | 22.67766788  21.20295511  25.75467463  27.5316263  21.83900751  28.10847798 |
| TRAIL | 26 | rs10164260  rs11081739  rs11618126  rs11657269  rs11699445  rs13185784  rs13278062  rs138987090  rs146783010  rs148051545  rs193112415  rs3136596  rs477251  rs57396456  rs62093514  rs62093947  rs6506939  rs7229599  rs72955191  rs73039026  rs747324  rs74778900  rs75928541  rs77451439  rs79287178  rs9952273 | 18  18  13  17  20  5  8  18  11  19  18  3  3  18  18  18  18  18  18  3  14  18  4  18  3  18 | 29060053  29583126  50826239  6319784  15750790  179694068  23082971  30366247  89260213  38602957  28835120  172228872  172162837  27945877  29230977  29660305  29312725  27785170  32367579  172160481  74689644  28086266  16401771  29399005  172294500  29575063 | G  G  G  G  G  G  G  G  G  C  C  G  C  C  C  C  C  C  G  C  C  C  G  G  G  C | A  A  A  A  T  A  T  A  A  T  T  A  T  T  T  T  T  T  A  A  T  T  A  A  A  T | -0.1018  -0.1411  -0.8908  -0.1188  -0.0746  -0.0846  -0.0801  0.7497  0.6016  0.3921  1.0421  -0.1147  -0.1497  0.5626  -1.0618  0.7596  -0.0927  -0.1431  -0.2386  0.2999  0.0855  -0.5906  -0.275  0.4322  0.4317  -0.864 | 0.0211  0.0202  0.1914  0.026  0.0161  0.0183  0.0157  0.0752  0.135  0.0848  0.0623  0.0209  0.0279  0.0518  0.0552  0.046  0.0169  0.027  0.0448  0.0635  0.0178  0.0532  0.0593  0.0369  0.0421  0.0499 | 1.51E-06  3.34E-12  1.46E-06  4.78E-06  3.27E-06  3.90E-06  3.57E-07  4.50E-23  4.83E-06  3.86E-06  2.15E-62  3.65E-08  7.94E-08  1.25E-27  6.86E-82  3.31E-61  4.17E-08  1.14E-07  1.27E-07  2.02E-06  1.61E-06  2.59E-28  4.24E-06  1.21E-31  9.12E-25  3.86E-69 | 0.071072  -0.0489704  0.308757  -0.00179007  -0.000245073  -0.0210488  -0.0221682  0.450851  0.19548  0.0319715  -0.0137625  0.033018  -0.0987725  -0.0689851  0.0697603  -0.015188  -0.0557075  -0.0185758  -0.0134398  0.226998  -0.0274814  0.0267897  0.00551057  -0.0510781  0.251126  0.173654 | 0.049793695  0.042032807  0.129803037  0.044821848  0.03324914  0.037425813  0.032399359  0.208043305  0.133514259  0.109442111  0.120373633  0.039406003  0.04582847  0.094362376  0.104180142  0.084808914  0.035452352  0.049476126  0.089633798  0.151040834  0.034817608  0.133924029  0.119063511  0.064764168  0.104278957  0.132102022 | 0.153485  0.243998  0.0173758  0.968143  0.994119  0.573834  0.493838  0.0302271  0.143163  0.770186  0.908975  0.402091  0.0311411  0.464739  0.503105  0.857871  0.116105  0.707326  0.880811  0.132867  0.429939  0.841452  0.963085  0.4303  0.0160308  0.188663 | 23.27719503  48.79229977  21.66090262  20.87786982  21.46969639  21.37167428  26.0294941  99.38923332  19.85857668  21.37971837  279.7967712  30.11856414  28.78957105  117.9614049  370.004739  272.6806049  30.08749694  28.09  28.36513473  22.30516709  23.07237091  123.2433857  21.50581972  137.188211  105.1477311  299.7963864 |
| TNFβ | 6 | rs10925040  rs116196280  rs148867298  rs753274  rs7629875  rs78296352 | 1  1  1  19  3  1 | 247622698  23047050  22716406  14436462  174385622  22821844 | C  G  C  C  G  G | T  T  T  T  A  T | -0.1755  -0.7179  -0.3157  0.1736  -0.3766  -1.2215 | 0.0373  0.1006  0.069  0.0371  0.0774  0.1366 | 2.67E-06  4.98E-13  4.52E-06  2.77E-06  1.37E-06  4.76E-21 | -0.0310804  0.0376048  0.0112988  -0.0161328  0.101788  -0.089286 | 0.033910595  0.112685541  0.06441577  0.032754045  0.074137667  0.083674641 | 0.359384  0.738595  0.860762  0.622335  0.169765  0.285944 | 22.13790798  50.92510642  20.93394035  21.89533642  23.67438522  79.96234906 |
| TNFα | 5 | rs10834997  rs111332265  rs115669577  rs79105320  rs8121916 | 11  5  4  8  20 | 26526948  150393107  124361448  18817360  12401325 | G  G  G  G  C | A  A  A  A  A | 0.1247  0.3766  -0.9889  -0.5605  -0.1306 | 0.0258  0.0754  0.1995  0.1179  0.0278 | 1.33E-06  6.63E-07  8.28E-07  3.59E-06  2.72E-06 | -0.022492  -0.000552818  0.170819  -0.171933  -0.0398521 | 0.035174635  0.071697103  0.171610257  0.129187603  0.037596508 | 0.522538  0.993848  0.319547  0.183229  0.289147 | 23.36111111  24.94697775  24.57078059  22.60079019  22.06971689 |
| SDF1α | 8 | rs10474392  rs12407262  rs13400104  rs139840550  rs149893336  rs4581824  rs482700  rs67689854 | 5  1  2  9  4  19  4  16 | 91494593  63820276  233810199  38688622  171232591  9185529  116067490  89625227 | G  G  G  G  G  G  G  C | A  A  A  A  A  T  A  A | -0.0962  -0.1179  0.0647  -0.1834  0.5034  0.0701  0.0893  0.0681 | 0.0178  0.0266  0.0189  0.0549  0.1081  0.0173  0.0203  0.0195 | 1.24E-06  3.99E-06  4.53E-06  3.79E-06  4.52E-06  3.05E-06  1.57E-06  3.07E-06 | 0.0163771  0.00607212  -0.0255163  0.0660126  0.0444333  -0.0238203  -0.0767804  0.0580017 | 0.037034406  0.046920085  0.045771088  0.090657812  0.134997162  0.033960735  0.036983594  0.049378932 | 0.658335  0.89703  0.577202  0.466521  0.742048  0.483049  0.0378878  0.240145 | 29.20855953  19.64555656  11.71884886  11.15973736  21.68577935  16.41889138  19.35133102  12.19621302 |
| SCGFβ | 16 | rs112346514  rs116924815  rs117716477  rs117865577  rs12480722  rs139413256  rs143829871  rs151194174  rs17876031  rs264162  rs34911860  rs4656185  rs4737732  rs73185877  rs7762066  rs78217154 | 19  19  12  19  20  7  3  7  5  18  1  1  8  12  6  8 | 12407988  51230733  104240958  51238105  20228904  145879644  47597245  20995778  176831119  10944026  80350715  169476326  66333628  103773440  95177967  101554072 | C  C  C  C  C  G  C  G  G  G  G  G  G  G  C  C | T  T  A  T  T  A  T  A  A  A  A  A  A  A  T  T | 0.3314  -0.6079  -0.8384  -0.2541  -0.1624  0.5377  0.1902  -0.4635  0.1514  -0.1097  0.3675  -0.205  0.1147  -0.5249  -0.1389  -0.3997 | 0.0711  0.0738  0.0841  0.0555  0.0355  0.1084  0.04  0.0942  0.0255  0.0234  0.0789  0.0256  0.0252  0.0711  0.0299  0.0864 | 2.37E-06  1.74E-16  1.34E-23  3.67E-06  4.72E-06  7.04E-07  1.90E-06  1.13E-06  2.25E-09  2.69E-06  3.24E-06  1.16E-15  4.68E-06  1.18E-13  3.50E-06  3.77E-06 | 0.041832  0.0302535  -0.0794098  -0.0266536  -0.00634915  0.119138  0.0866537  0.0102199  0.0305486  -0.012793  -0.035029  0.0124788  -0.0263484  -0.126189  -0.0553937  -0.159683 | 0.097888104  0.097743695  0.139554809  0.087402717  0.051265337  0.089621136  0.066869239  0.120285042  0.035567069  0.032884359  0.191480619  0.034544598  0.03871442  0.104907132  0.035673821  0.113846724 | 0.669128  0.756927  0.569341  0.760403  0.901435  0.183732  0.195021  0.93229  0.390395  0.697254  0.854847  0.717922  0.496135  0.229029  0.120475  0.160732 | 21.72530122  67.85026733  99.38264424  20.96154858  20.92740329  24.60489458  22.610025  24.21016066  35.25099577  21.97766455  21.69505125  64.12506104  20.71694696  54.50218883  21.58053042  21.40133236 |
| SCF | 9 | rs113127926  rs13412535  rs1557570  rs1568119  rs1942355  rs4841899  rs635634  rs78666213  rs80271436 | 14  2  1  8  18  9  9  4  9 | 98437511  224874874  169507844  33243197  69361739  137424412  136155000  180138649  135897770 | C  G  G  C  C  C  C  G  G | A  A  T  T  T  T  T  T  A | -0.1982  0.1067  -0.1186  0.5906  0.0716  0.1004  0.1032  0.2744  0.237 | 0.042  0.0213  0.017  0.1129  0.0157  0.0178  0.0191  0.0576  0.0485 | 2.27E-06  6.04E-07  2.74E-12  1.24E-07  4.70E-06  1.78E-08  6.74E-08  2.59E-06  9.95E-07 | -0.0969268  0.107429  0.010233  0.349524  0.0325306  -0.0644585  -0.0848675  0.00454505  -0.0916416 | 0.065495351  0.03853527  0.034523128  0.171155118  0.032637959  0.0349881  0.041864604  0.09299356  0.074947268 | 0.138899  0.0053066  0.766917  0.0411369  0.318905  0.0654316  0.042643  0.961019  0.221425 | 22.26941043  25.09398488  48.67114187  27.36522362  20.79824739  31.81466986  29.19393657  22.69463735  23.87883941 |
| IL-16 | 10 | rs117217798  rs117916513  rs1255143  rs12765671  rs144691581  rs1801020  rs4253283  rs4513633  rs4778636  rs9706053 | 17  11  10  10  15  5  4  4  15  12 | 31483233  121264274  130052200  106684169  96953325  176836532  187165211  113570639  81591639  66376310 | C  G  C  G  G  G  C  C  G  C | T  A  T  A  A  A  T  A  A  T | 0.2036  0.502  -0.1306  0.6023  -0.4882  -0.1733  -0.146  0.2239  0.7272  -0.4582 | 0.0444  0.0986  0.0242  0.1318  0.0967  0.0272  0.0262  0.0453  0.0633  0.0932 | 4.15E-06  3.79E-07  7.10E-08  4.84E-06  4.20E-07  4.53E-10  1.75E-08  7.44E-07  1.11E-30  7.01E-07 | 0.0167644  0.25885  -0.023373  0.0708242  0.0384551  0.0182773  0.00203533  0.062373  0.00403687  0.100403 | 0.066697255  0.146251994  0.032888701  0.10133307  0.142287747  0.037596029  0.034934139  0.048044827  0.0560971  0.113339349 | 0.801543  0.0767454  0.477289  0.484599  0.786958  0.626861  0.95354  0.19421  0.942632  0.375692 | 21.02759516  25.92111056  29.12430845  20.883097  25.48840164  40.59376352  31.05296894  24.42934277  131.9776285  24.17009431 |
| RANTES | 10 | rs112072646  rs147509526  rs2251660  rs4940620  rs62438851  rs7000423  rs72793342  rs74472919  rs75613039  rs818452 | 2  19  17  18  6  8  16  13  11  6 | 53444393  15776330  34252537  61971111  145230309  111053649  30548352  82200650  129576583  152915796 | G  C  C  G  G  C  G  C  C  C | A  T  A  A  A  T  A  T  T  T | -0.4286  0.358  -0.1829  0.2494  0.1957  0.1318  0.1487  -0.3313  -0.37  -0.2381 | 0.0862  0.0717  0.0359  0.054  0.0414  0.0253  0.0308  0.0605  0.081  0.0505 | 6.48E-07  6.93E-07  3.83E-07  3.54E-06  2.33E-06  1.82E-07  1.48E-06  3.97E-08  4.81E-06  2.36E-06 | 0.09128  -0.0359109  0.00979864  0.00612041  0.0621425  -0.0153582  0.0533671  0.118949  0.0405806  -0.0361448 | 0.099090821  0.141790031  0.045518228  0.069130359  0.048326187  0.034684671  0.040628909  0.102059283  0.096113874  0.062366352 | 0.356959  0.800061  0.829558  0.929452  0.198479  0.657914  0.189006  0.243821  0.672869  0.562214 | 24.72235292  24.93031362  25.95604472  21.33071331  22.34503139  27.13874611  23.30883159  29.98693805  20.86572169  22.22982453 |
| PDGFbb | 18 | rs116154010  rs116445074  rs11766649  rs11916118  rs12289510  rs12990266  rs13024765  rs13412535  rs192607922  rs2324229  rs28406863  rs35859699  rs4965869  rs72777070  rs73162807  rs9806745  rs9936075  rs9941733 | 2  5  7  3  11  2  2  2  2  6  15  4  15  2  3  15  16  20 | 224556644  51534600  144839247  116912189  124947051  224306859  225180219  224874874  225747138  83918131  102086276  112184751  101990320  9798877  146474790  102010311  7321909  374061 | C  G  G  G  G  G  C  G  G  C  G  G  C  G  C  C  G  G | T  T  A  A  A  A  T  A  T  T  T  A  T  T  A  A  A  A | -0.3213  -0.2931  -0.0908  -0.0889  0.078  -0.2363  0.1014  -0.3352  0.244  -0.0894  0.2089  0.3952  -0.184  0.1069  0.2391  -0.1162  0.0782  -0.1161 | 0.0663  0.0587  0.0196  0.0194  0.0158  0.0342  0.0158  0.0214  0.0498  0.0161  0.0382  0.0842  0.0181  0.02  0.0499  0.0163  0.0164  0.0228 | 1.23E-06  3.11E-07  3.53E-06  4.93E-06  7.69E-07  3.18E-12  1.14E-10  2.46E-55  1.16E-06  3.48E-08  4.78E-08  2.07E-06  5.66E-24  8.98E-08  1.74E-06  1.10E-12  1.76E-06  3.31E-07 | 0.136279  0.104225  0.00361434  -0.0373291  -0.0110913  0.00679025  -0.0200473  0.107429  -0.139791  -0.0791511  0.0111699  -0.00231907  0.0305727  0.0247343  0.199908  0.0312914  -0.0275313  0.040339 | 0.127023782  0.135003988  0.037041289  0.050730925  0.032530421  0.094770631  0.032826252  0.03853527  0.161742009  0.033540755  0.095868289  0.109512392  0.036689055  0.043579409  0.114335001  0.035250625  0.034267703  0.043479085 | 0.283333  0.440106  0.922269  0.461837  0.73314  0.942881  0.541392  0.0053066  0.387432  0.0182824  0.907246  0.983105  0.40468  0.570328  0.0803877  0.374711  0.421733  0.353522 | 23.4852071  24.93190333  21.46147439  20.99907004  24.37109438  47.73921036  41.1871495  245.3468425  24.00606442  30.83353266  29.90543708  22.02975609  103.3423888  28.569025  22.95926924  50.82027927  22.73661511  25.92953601 |
| MIP1β | 44 | rs10491120  rs111942332  rs112078619  rs113010081  rs113877493  rs116237296  rs117120228  rs117394484  rs117453826  rs117620244  rs117715247  rs141040281  rs141793738  rs149949150  rs17138331  rs17548645  rs17693183  rs2190980  rs2305097  rs2531756  rs2673050  rs281749  rs35933743  rs4683315  rs4795162  rs4796072  rs60516659  rs6505501  rs7215719  rs7221878  rs72791296  rs72799710  rs73074316  rs74810984  rs76582507  rs76583883  rs76776296  rs76842834  rs76960253  rs79091774  rs80007108  rs80322601  rs9330240  rs9850846 | 17  17  17  3  17  1  17  17  17  17  17  3  17  17  7  17  17  17  17  3  3  8  17  3  17  17  17  17  17  17  5  5  3  10  9  21  7  17  17  3  17  17  17  3 | 34988444  34818508  33836531  46457412  34812273  87045516  33780205  34363701  35132809  33648381  33978654  47006232  35386610  35118636  7866368  33732932  34964290  32996753  35550448  45856364  45739807  108638645  32970484  46752153  35236530  33652270  34403297  34347238  35193419  36191133  120950050  123161665  48406223  129674466  37510072  47356359  115128487  34883848  34088656  45906878  35012096  33638697  34974689  45114682 | G  G  G  C  C  G  G  C  G  C  G  G  G  G  G  C  G  G  C  C  G  C  G  G  G  G  G  C  G  C  C  C  G  C  G  G  G  C  C  C  C  C  C  G | A  T  A  T  T  A  A  T  A  T  A  A  A  A  A  T  A  A  A  A  T  T  T  A  A  T  A  T  A  T  T  T  A  T  A  T  A  T  T  A  A  T  T  A | -0.3001  0.4727  -0.2675  0.5954  0.6124  -0.5437  0.3405  0.421  0.5774  0.3528  0.351  0.1045  -0.1855  -0.2249  0.1391  0.1472  0.5795  0.1067  -0.0932  0.1048  -0.1314  -0.0799  0.126  0.1347  -0.1261  0.1149  -0.2691  0.1556  -0.0786  0.3045  -0.2369  0.1014  0.1436  -0.2206  -0.3175  0.2317  -0.2997  0.4206  -0.5233  0.4606  -0.2259  0.1952  0.4745  -0.072 | 0.0318  0.0573  0.0486  0.0236  0.0218  0.1115  0.0743  0.0783  0.0593  0.0495  0.059  0.021  0.0392  0.0478  0.0295  0.0306  0.0795  0.0167  0.0189  0.0221  0.0161  0.0171  0.0239  0.0236  0.0158  0.0177  0.0248  0.0191  0.0168  0.0463  0.0466  0.0218  0.0216  0.0474  0.0677  0.0511  0.0598  0.0472  0.0585  0.0751  0.031  0.0381  0.046  0.0157 | 5.15E-21  1.70E-16  5.46E-08  3.85E-140  1.62E-173  7.23E-07  4.27E-06  7.69E-08  5.07E-22  1.87E-12  3.09E-09  6.26E-07  1.87E-06  2.54E-06  2.26E-06  1.38E-06  8.93E-13  1.43E-10  8.22E-07  1.99E-06  3.14E-16  3.17E-06  1.50E-07  8.97E-09  1.14E-15  8.12E-11  3.64E-27  3.71E-16  2.99E-06  7.37E-11  3.78E-07  3.21E-06  2.80E-11  1.96E-06  3.26E-06  4.99E-06  5.55E-07  7.33E-19  5.45E-19  8.83E-10  2.73E-13  2.56E-07  5.84E-25  4.48E-06 | 0.0549417  -0.0620999  -0.0308503  0.0130561  -0.0583724  -0.168571  0.0169156  -0.112432  0.283124  0.0468761  -0.105031  0.0410414  -0.0528944  0.0811563  -0.0443997  -0.124512  -0.0436809  0.0240245  0.0598308  0.0186732  0.0221743  0.033664  0.0223762  -0.0420009  -0.0196394  0.0616292  -0.0821988  0.00553744  0.0144377  0.0485295  0.0840615  -0.065094  -0.0771082  0.10828  -0.0463471  -0.118669  -0.061328  0.0790387  0.0844713  -0.0954776  0.0631966  0.078976  0.0144978  0.0233316 | 0.052915118  0.092891019  0.097567526  0.051905831  0.059990415  0.158125076  0.150453712  0.118669104  0.129202373  0.147959262  0.105719402  0.043094379  0.116749382  0.172067051  0.050292139  0.059596403  0.131849658  0.033372104  0.041005932  0.055840745  0.032840264  0.034801372  0.049146744  0.061158191  0.032951084  0.041214412  0.054386918  0.042139064  0.035018674  0.074143443  0.08005507  0.041945599  0.072754356  0.126586262  0.135903294  0.082775622  0.084067623  0.070177068  0.116006601  0.095626571  0.068649  0.082332723  0.077082142  0.032743268 | 0.299131  0.503799  0.751855  0.801401  0.330539  0.286396  0.910482  0.343414  0.0284285  0.751382  0.320472  0.340914  0.650506  0.637173  0.377325  0.0366849  0.740423  0.471588  0.144544  0.738077  0.499538  0.333385  0.648898  0.492235  0.551164  0.134828  0.130694  0.895452  0.68013  0.512767  0.293697  0.120694  0.289216  0.392338  0.733081  0.15168  0.465691  0.260049  0.466516  0.318065  0.357272  0.337443  0.850812  0.476117 | 89.05898699  68.05530124  30.29528442  636.4930336  789.1460315  23.77765006  21.00180419  28.9095058  94.80782257  50.79801653  35.39241597  24.76247166  22.39317602  22.13722186  22.23362252  23.14050152  53.13401369  40.8221521  24.31690042  22.48733646  66.60993017  21.83239287  27.79363106  32.57700733  63.69656305  42.13990233  117.7400007  66.36704038  21.88903061  43.25263914  25.84391405  21.63530006  44.1978738  21.65979455  21.99429886  20.55939201  25.11719388  79.40622307  80.0183768  37.61559997  53.1017794  26.24881339  106.4037098  21.03127916 |
| MIP1α | 8 | rs10835056  rs12690897  rs184154340  rs34771762  rs57786342  rs60198979  rs6900267  rs7232268 | 11  7  11  2  14  22  6  18 | 26697017  85346177  80502036  201412655  69260028  43646704  380341  67768914 | G  G  G  G  G  G  C  G | T  A  A  A  A  A  A  A | -0.1194  -0.1248  -0.331  -0.249  -0.1314  0.2146  0.2429  -0.2821 | 0.0254  0.0262  0.0693  0.0523  0.0285  0.0458  0.0519  0.0599 | 2.60E-06  2.11E-06  1.86E-06  2.13E-06  4.06E-06  2.62E-06  2.89E-06  2.55E-06 | 0.0228767  -0.00886365  -0.03507  -0.00719344  -0.00368629  0.0841168  -0.0274374  0.0204967 | 0.037385329  0.03723777  0.080102479  0.064608792  0.040435652  0.061193299  0.081335323  0.087976738 | 0.540593  0.811859  0.661521  0.911348  0.927362  0.169253  0.735863  0.815778 | 22.09740219  22.68958685  22.8133739  22.66706638  21.25695291  21.95474915  21.9038428  22.17953963 |
| MIG | 14 | rs111607343  rs11177248  rs112337562  rs112861654  rs117831247  rs139010077  rs1796086  rs41272086  rs55876513  rs5752128  rs62562991  rs6679677  rs77086208  rs816960 | 19  12  14  21  10  3  7  6  4  22  9  1  14  13 | 897855  68875886  93131570  43599172  68501839  170336148  70648715  161008646  76883698  25718623  98736059  114303808  70619491  108522521 | G  G  G  G  C  C  C  G  G  C  G  C  C  C | A  A  T  A  T  T  T  A  T  T  A  A  T  T | 0.521  -0.3073  0.37  0.2765  0.8334  -0.4322  0.2096  0.2226  -0.166  0.1685  -0.6236  -0.162  -0.3226  0.1224 | 0.1119  0.067  0.0796  0.0529  0.1754  0.095  0.0403  0.0415  0.0255  0.0369  0.126  0.0329  0.0698  0.0244 | 2.83E-06  4.45E-06  2.98E-06  1.81E-07  2.16E-06  3.55E-06  2.23E-07  7.43E-08  8.23E-11  4.34E-06  8.40E-07  8.86E-07  3.83E-06  5.01E-07 | -0.194609  -0.0361348  0.00144573  -0.0454209  0.00302957  -0.201517  0.0158389  -0.0264318  0.0818017  0.019794  0.214262  0.00134804  -0.235165  0.0446843 | 0.096300293  0.066207952  0.166933607  0.057826013  0.150832113  0.14408172  0.057986388  0.052816177  0.037858544  0.055914857  0.118074891  0.053511123  0.125821882  0.040315913 | 0.0432947  0.585219  0.99309  0.432175  0.983975  0.161924  0.784739  0.616759  0.030717  0.723337  0.0695811  0.979902  0.0616185  0.267709 | 21.67780341  21.03659835  21.60614631  27.31988879  22.57604251  20.6977108  27.05032357  28.77094498  42.3775471  20.85196936  24.49464349  24.24589573  21.36081806  25.16420317 |
| MCSF | 8 | rs116274860  rs117867915  rs12962919  rs145778765  rs56367447  rs62294910  rs78296352  rs9387100 | 3  18  18  20  8  3  1  6 | 148392817  42210043  75778756  3182002  3871527  182198339  22821844  113102954 | G  C  C  C  C  G  G  C | T  T  T  T  T  A  T  T | -0.819  -0.5272  -0.3052  0.7993  0.4967  -0.3431  -0.527  0.1352 | 0.1741  0.1098  0.0662  0.1689  0.0883  0.0691  0.1112  0.0292 | 2.74E-06  1.61E-06  4.65E-06  2.20E-06  1.72E-08  6.82E-07  1.05E-06  4.07E-06 | -0.120274  -0.221367  0.0425258  0.0470704  0.114793  -0.0107336  -0.089286  0.0228749 | 0.129702957  0.18365481  0.054530841  0.114347585  0.08688251  0.070927005  0.083674641  0.034230889 | 0.353769  0.228071  0.43548  0.680601  0.18642  0.879713  0.285944  0.503972 | 22.12943171  23.05399119  21.25460702  22.39547751  31.6422176  24.65388361  22.46009847  21.43816851 |
| MCP3 | 3 | rs10892381  rs62492260  rs73669117 | 11  8  8 | 119400817  10476528  27835190 | C  G  G | T  T  A | -0.2412  0.2788  0.6238 | 0.0476  0.058  0.131 | 3.56E-07  1.54E-06  2.56E-06 | -0.0127657  -0.0609002  0.209745 | 0.036522549  0.05118907  0.137979665 | 0.726692  0.23416  0.128482 | 25.67678836  23.10625446  22.67504458 |
| MCP1 | 18 | rs10744620  rs10888395  rs111995966  rs12073356  rs12075  rs145155829  rs146522229  rs2036297  rs2228467  rs2712431  rs56212190  rs7197349  rs7517040  rs762787  rs7632755  rs78629618  rs79457566  rs9317045 | 12  1  2  1  1  1  19  3  3  3  1  16  1  3  3  3  3  13 | 3739094  150762171  109174969  208007848  159175354  44165646  47798480  46172903  42906116  128316890  42168539  78687219  158859133  46488936  46332382  46880130  45674359  59630038 | C  C  G  G  G  C  C  G  C  C  C  G  G  C  G  C  G  C | T  T  T  A  A  T  T  A  T  A  T  A  A  T  A  T  A  A | -0.0788  0.0814  -0.1452  0.1426  -0.2185  0.2153  0.5976  -0.119  0.2637  0.0787  -0.181  -0.0968  0.0987  0.1371  -0.2938  -0.1188  0.2113  -0.1134 | 0.0161  0.0163  0.031  0.0311  0.0155  0.0463  0.1177  0.016  0.0291  0.0172  0.0373  0.0206  0.0191  0.0283  0.0316  0.0259  0.0461  0.0236 | 9.91E-07  5.98E-07  2.53E-06  4.17E-06  1.44E-44  3.72E-06  3.56E-07  1.09E-13  9.19E-20  4.76E-06  9.85E-07  2.62E-06  2.44E-07  1.22E-06  1.18E-20  4.23E-06  3.95E-06  1.52E-06 | 0.0135363  0.00886696  -0.0169496  -0.1061  -0.0585035  0.0624563  0.0726502  0.0103922  -0.00415055  0.0513862  -0.151297  0.0275852  -0.00786988  0.0581116  -0.00643944  0.0390429  -0.087329  0.0347964 | 0.033939624  0.033620366  0.127669539  0.067068383  0.032794475  0.090145973  0.150922253  0.034304098  0.067422521  0.034870908  0.080490576  0.050024455  0.037408589  0.062888425  0.06412677  0.057238236  0.075437898  0.044563302 | 0.690015  0.791982  0.894382  0.113657  0.0744328  0.488413  0.63025  0.761933  0.950913  0.140586  0.0601509  0.581336  0.833374  0.355464  0.920013  0.495168  0.247016  0.434902 | 23.95524864  24.93868794  21.93864724  21.0241416  198.7190427  21.62350433  25.77912972  55.31640625  82.11722819  20.93594511  23.54721158  22.08087473  26.70346208  23.46940279  86.4429178  21.03940013  21.0086015  23.08883941 |
| IL-12p70 | 11 | rs12199215  rs145023524  rs17229494  rs282258  rs3025021  rs4349809  rs71361173  rs7754905  rs7757246  rs782107  rs79121401 | 6  6  21  2  6  6  18  6  6  12  11 | 44026914  43819046  38928100  224914800  43749163  43924830  73712405  44150182  44003982  58833530  78697129 | C  G  G  C  C  G  G  G  C  G  C | T  A  A  T  T  T  T  A  T  A  T | -0.1278  -0.279  0.1172  -0.073  -0.0898  -0.3777  -0.111  0.1029  -0.139  -0.075  -0.5548 | 0.0192  0.0394  0.0257  0.0156  0.0188  0.0159  0.0239  0.019  0.0285  0.0156  0.1206 | 5.11E-11  1.54E-12  4.93E-06  3.21E-06  2.20E-06  2.56E-124  3.06E-06  4.28E-08  1.97E-06  1.60E-06  4.24E-06 | 0.018952  -0.231854  0.0402668  -0.000792005  0.0221488  -0.0315038  -0.00559556  0.0431577  -0.00323413  -0.0524145  -0.160766 | 0.037128727  0.19525863  0.058532624  0.032884074  0.036546291  0.032592451  0.046721995  0.037903061  0.052024477  0.032663129  0.134826877 | 0.609743  0.235062  0.491492  0.980785  0.544483  0.333745  0.904671  0.254856  0.950431  0.10856  0.233109 | 44.30566406  50.14365225  20.79643901  21.89760026  22.81586691  564.2865789  21.57000053  29.33077562  23.78701139  23.11390533  21.16305152 |
| IP10 | 13 | rs10809307  rs113831257  rs11626201  rs143799975  rs188759467  rs34383175  rs397816  rs75970138  rs7645625  rs7690978  rs79848609  rs8112909  rs9450351 | 9  4  14  4  5  8  22  9  3  4  15  19  6 | 11045908  76159521  36980700  76807015  73103247  145584694  22728326  122576276  146574037  75991844  87316165  46413408  86624320 | C  G  C  G  T  C  C  G  G  G  C  G  C | T  A  A  A  A  T  T  A  T  A  A  A  T | -0.1305  -0.3592  -0.1162  0.7984  -0.4504  0.3153  -0.1237  0.485  0.1086  -0.1247  -0.2603  0.1426  0.2768 | 0.0282  0.0644  0.0245  0.1637  0.0964  0.0657  0.0249  0.104  0.0237  0.0259  0.0537  0.0299  0.0489 | 3.64E-06  2.53E-08  1.93E-06  1.00E-06  3.22E-06  1.51E-06  7.90E-07  1.53E-06  4.41E-06  1.46E-06  8.75E-07  1.94E-06  1.48E-08 | 0.00582432  0.0799785  0.00740115  0.224296  0.0140446  0.134412  0.00872109  -0.133515  0.00348095  0.0411836  0.0765414  0.0161195  0.01695 | 0.035353629  0.086679928  0.03353781  0.148478547  0.087128137  0.087560913  0.032926312  0.179058591  0.033071006  0.037558514  0.079859583  0.04054725  0.066966174 | 0.869145  0.35617  0.825341  0.130883  0.87194  0.124767  0.791112  0.455879  0.916172  0.272852  0.337836  0.690963  0.800181 | 21.41523314  31.1100652  22.49469388  23.78722047  21.82944509  23.03123371  24.6797471  21.74787352  20.99727608  23.18106468  23.49631548  22.74556213  32.0416191 |
| IL-18 | 17 | rs10414578  rs115267715  rs116383510  rs116656892  rs11700536  rs117266781  rs144841621  rs17229943  rs1852105  rs1979967  rs2729385  rs385076  rs4482818  rs658805  rs71478720  rs78623212  rs78716465 | 19  5  5  5  21  7  10  5  7  15  11  2  4  6  11  7  20 | 55146070  68535015  2545650  68186028  44558687  41301020  71681557  68682536  63725595  79659613  57262993  32489851  65928497  70909073  112009605  103307627  40643726 | C  C  C  C  C  C  C  C  C  C  G  C  G  G  C  C  G | T  T  A  T  T  T  T  A  T  T  A  T  A  A  T  T  A | 0.1771  -0.4508  0.5426  -0.5298  -0.1156  -0.6841  -0.518  0.312  -0.3036  -0.1402  -0.1231  0.2432  -0.1286  -0.1226  0.2669  -0.8705  -0.3265 | 0.035  0.08  0.1056  0.0925  0.025  0.1468  0.1141  0.0463  0.0661  0.0286  0.0262  0.0248  0.0244  0.0244  0.0276  0.1778  0.0682 | 4.16E-07  1.72E-08  3.00E-07  1.05E-08  4.21E-06  3.15E-06  3.81E-06  1.62E-11  4.32E-06  9.45E-07  3.79E-06  1.66E-22  1.45E-07  4.94E-07  3.71E-22  6.71E-07  1.63E-06 | 0.0165557  0.0241038  -0.116154  -0.0490387  0.0253704  -0.186836  0.270665  -0.0358095  0.00293093  -0.00817282  0.0207349  -0.0853437  -0.0402104  -0.0257418  -0.00649016  -0.143731  -0.0907226 | 0.051461058  0.171633133  0.147504752  0.105398651  0.033086548  0.148780594  0.261177964  0.073960759  0.071181438  0.038913994  0.035045713  0.034028088  0.033551294  0.034571807  0.037202517  0.105753585  0.087765207 | 0.74767  0.888314  0.431013  0.641739  0.443207  0.209195  0.300051  0.628266  0.967156  0.83365  0.554083  0.0121404  0.230732  0.45652  0.861508  0.174111  0.301278 | 25.6036  31.753225  26.40169091  32.80503024  21.381376  21.71636186  20.6104859  45.40955082  21.09602422  24.03056384  22.07565119  96.16649324  27.77808385  25.24650632  93.51450588  23.97033136  22.91910329 |
| IL-17 | 10 | rs117029961  rs117556572  rs1530455  rs17106604  rs17282552  rs184080173  rs187475560  rs62191444  rs78296352  rs78612928 | 10  13  3  14  2  12  4  20  1  4 | 37436581  105088917  122854899  78379156  207973815  77725204  161274563  373667  22821844  29813914 | G  C  C  C  C  C  C  G  G  C | A  T  T  T  T  T  T  T  T  T | -0.4585  0.5102  -0.108  -0.1129  0.2001  -0.2384  0.2434  0.1136  -0.3027  -0.1037 | 0.1015  0.1099  0.0173  0.0225  0.0405  0.0471  0.052  0.0247  0.0646  0.0222 | 4.94E-06  3.28E-06  4.87E-10  6.37E-07  8.21E-07  4.19E-07  3.29E-06  4.22E-06  4.27E-06  2.62E-06 | 0.0592098  -0.201057  0.0228385  -0.0382877  0.0581911  -0.0950222  0.0735513  -0.049165  -0.089286  0.0940267 | 0.168469206  0.123401017  0.033737848  0.051467625  0.091703198  0.065291585  0.175667365  0.043633779  0.083674641  0.041677962 | 0.725245  0.10325  0.498444  0.456926  0.525716  0.145572  0.675438  0.259842  0.285944  0.0240688 | 20.40546968  21.55189804  38.97223429  25.17809383  24.41091907  25.61950226  21.90960059  21.15255126  21.95633285  21.81984011 |
| IL-13 | 12 | rs117795020  rs12623722  rs139083458  rs2370048  rs27949  rs6799107  rs7073807  rs75438658  rs75995699  rs7757246  rs9296421  rs9472168 | 9  2  5  3  5  3  10  6  6  6  6  6 | 90084152  23178683  26160518  132960738  58550823  127057018  69153428  44018321  5140856  44003982  43867233  43928985 | G  G  C  G  C  C  C  C  G  C  G  G | A  A  T  A  T  T  T  T  A  T  T  A | 0.3522  0.1185  -0.9902  0.1235  0.1168  0.1459  -0.1682  0.343  -0.3319  -0.2171  -0.1746  -0.4244 | 0.0716  0.0258  0.2107  0.0266  0.0252  0.0301  0.0356  0.0625  0.0698  0.0423  0.0349  0.0248 | 9.86E-07  4.19E-06  2.81E-06  3.35E-06  3.43E-06  1.25E-06  2.37E-06  4.12E-08  2.64E-06  3.53E-07  6.30E-07  1.08E-65 | -0.158972  0.0192671  0.107427  -0.00193959  0.0439044  0.039603  0.0327495  0.132805  0.0743804  -0.00323413  0.0289123  -0.0328784 | 0.120842867  0.035571636  0.123583925  0.036482941  0.034962106  0.039995416  0.052195067  0.158778675  0.105455017  0.052024477  0.054937755  0.033448213 | 0.188333  0.588065  0.384703  0.957601  0.209199  0.322082  0.530368  0.402921  0.480606  0.950431  0.598698  0.325625 | 24.19650604  21.09586263  22.08598499  21.55612245  21.48248929  23.49511595  22.32297058  30.118144  22.61016125  26.34140357  25.02866151  292.8514568 |
| IL-10 | 11 | rs10457128  rs10493718  rs11206302  rs2086656  rs282258  rs3025021  rs4349809  rs465757  rs6085948  rs6458375  rs7088799 | 6  1  1  4  2  6  6  20  20  6  10 | 106017976  83062933  54673943  60498473  224914800  43749163  43924830  15580283  7233350  44202352  65016174 | G  C  C  C  C  C  G  G  G  C  G | A  A  T  T  T  T  T  A  A  T  T | 0.0865  0.11  0.1189  0.0789  -0.0992  -0.0947  -0.2853  -0.084  -0.098  -0.0946  0.0852 | 0.0172  0.0222  0.0251  0.0171  0.0162  0.0195  0.0165  0.0174  0.0202  0.0198  0.0167 | 5.24E-07  7.16E-07  2.20E-06  3.78E-06  1.00E-09  1.46E-06  5.77E-67  1.17E-06  1.25E-06  2.07E-06  3.23E-07 | -0.035245  -0.00406512  -0.00989201  -0.0348182  -0.000792005  0.0221488  -0.0315038  -0.00655996  -0.0418224  -0.0049323  -0.0488009 | 0.034003441  0.038855421  0.060406431  0.035616647  0.032884074  0.036546291  0.032592451  0.035498131  0.038621077  0.038957773  0.032917116 | 0.299963  0.916676  0.869922  0.328281  0.980785  0.544483  0.333745  0.853388  0.278857  0.899252  0.138197 | 25.29154273  24.55157861  22.43965969  21.28931979  37.49672306  23.58472058  298.9755372  23.30558859  23.53690815  22.82716049  26.02832658 |
| IL-8 | 4 | rs11634944  rs12075  rs141926526  rs2673604 | 15  1  7  8 | 25183093  159175354  32848640  133411607 | C  G  C  C | T  A  A  A | 0.1214  -0.12  0.6149  0.1266 | 0.0252  0.0236  0.1308  0.0255 | 1.29E-06  3.88E-07  2.57E-06  7.02E-07 | 0.0703312  -0.0585035  -0.0103308  0.0339684 | 0.033883334  0.032794475  0.083488692  0.036127037 | 0.0379229  0.0744328  0.901522  0.34709 | 23.20792391  25.85463947  22.10006231  24.6483045 |
| IL-6 | 4 | rs1333040  rs13412535  rs73273528  rs76856708 | 9  2  20  16 | 22083404  224874874  50431113  80729043 | C  G  C  C | T  A  T  T | -0.0738  0.1164  -0.2672  -0.3289 | 0.0158  0.0215  0.0553  0.07 | 3.17E-06  7.34E-08  9.58E-07  2.61E-06 | 0.0580991  0.107429  -0.0918727  -0.309493 | 0.032886753  0.03853527  0.091147232  0.088782157 | 0.0772883  0.0053066  0.313474  0.0004903 | 21.81717673  29.31089237  23.34654637  22.07657347 |
| IL1ra | 6 | rs1054402  rs11627423  rs12121840  rs2809154  rs61335305  rs9623661 | 9  14  1  13  15  22 | 119163509  33200623  165541642  84727524  66453074  43093376 | C  C  C  C  C  C | T  A  T  T  A  T | -0.1311  -0.1171  -0.2692  0.1786  -0.4453  0.1966 | 0.027  0.0247  0.0571  0.0388  0.0908  0.0426 | 1.13E-06  2.12E-06  2.43E-06  3.74E-06  1.00E-06  3.86E-06 | -0.0108874  0.0206869  -0.0277638  0.0655244  -0.140418  -0.00999153 | 0.037908225  0.033130286  0.069077949  0.04177857  0.121682933  0.057956763 | 0.773956  0.532358  0.687743  0.116794  0.248514  0.863126 | 23.57641975  22.47604452  22.22684877  21.18846317  24.05103073  21.29844167 |
| IL-1β | 6 | rs143319329  rs1942793  rs4786740  rs61335305  rs62015704  rs9898641 | 7  18  16  15  16  17 | 128139459  71104371  5675198  66453074  7467907  57571033 | C  G  C  C  G  C | T  T  A  A  A  T | -0.2801  -0.0717  -0.0845  -0.2966  -0.1082  0.2032 | 0.0715  0.0187  0.0202  0.0724  0.0283  0.0454 | 2.00E-06  4.98E-06  4.67E-06  1.90E-06  2.09E-06  3.59E-06 | -0.0493409  0.0544567  0.0134018  -0.140418  -0.0234064  0.0135564 | 0.632721  0.0963487  0.690276  0.248514  0.63371  0.69924 | 0.1032451  0.0327496  0.0336323  0.1216829  0.0491203  0.0350887 | 15.34666927  14.70127828  17.49889717  16.78282867  14.61778771  20.03252537 |
| HGF | 7 | rs11060254  rs150322232  rs1698249  rs2003620  rs3748034  rs5745687  rs62481625 | 12  7  14  7  4  7  7 | 129815569  7930374  84356186  134479484  3446091  81359051  155987460 | G  G  C  C  G  C  C | A  A  A  T  T  T  T | 0.08  -0.2104  0.1698  -0.2279  -0.1495  0.3072  -0.1091 | 0.0167  0.0463  0.0372  0.0489  0.0234  0.0406  0.0225 | 1.58E-06  4.89E-06  4.09E-06  2.83E-06  1.81E-10  2.75E-14  1.18E-06 | 0.0516347  -0.025279  -0.016386  0.0663502  -0.00229087  -0.059692  0.0144544 | 0.034039182  0.113231842  0.058160845  0.071789918  0.046551818  0.065486023  0.038196086 | 0.129287  0.823341  0.778146  0.355368  0.960751  0.36202  0.705114 | 22.94811574  20.65044853  20.83480749  21.7205557  40.81790123  57.25195952  23.51172346 |
| IL-9 | 6 | rs41294750  rs4880409  rs61867538  rs7232268  rs7242404  rs76963786 | 1  10  11  18  18  12 | 53550640  134330220  1524506  67768914  12741267  32039757 | C  C  C  G  G  C | T  T  T  A  A  T | -0.3514  0.3355  -0.3566  -0.2759  0.1228  0.2865 | 0.0748  0.0723  0.0774  0.0587  0.0264  0.0557 | 2.37E-06  3.50E-06  3.93E-06  2.53E-06  3.27E-06  4.50E-07 | 0.086602  -0.162983  0.0217759  0.0204967  -0.033448  0.0490238 | 0.097767132  0.101413649  0.089561184  0.087976738  0.036709014  0.055958773 | 0.375726  0.10803  0.807897  0.815778  0.362208  0.380992 | 22.06989762  21.53319406  21.22661565  22.09160139  21.6365932  26.4568943 |
| IL-7 | 10 | rs117509142  rs141425475  rs144701438  rs17091524  rs28793375  rs4320361  rs75904417  rs77981494  rs78346957  rs9296421 | 8  5  18  14  8  6  2  16  10  6 | 87134083  17679165  63960405  56948759  41415618  43928511  168653321  17544866  126903513  43867233 | C  C  G  C  C  G  C  C  G  G | T  T  A  T  T  T  A  T  A  T | 0.327  0.4781  0.4819  -0.4924  -0.1638  0.3245  0.1698  0.5178  -0.4588  -0.169 | 0.0688  0.1016  0.0989  0.1013  0.0361  0.0249  0.0349  0.1064  0.1007  0.0355 | 1.99E-06  2.53E-06  9.75E-07  1.91E-06  4.46E-06  6.87E-39  1.16E-06  1.07E-06  4.51E-06  2.10E-06 | 0.0321165  0.108095  0.0678873  -0.0371215  0.0206831  0.0289413  0.0372945  0.0123492  0.0806881  0.0289123 | 0.100418981  0.106015925  0.102596856  0.096232073  0.048701063  0.033150426  0.052888278  0.113561414  0.123267505  0.054937755 | 0.749101  0.307913  0.50817  0.699682  0.671059  0.382647  0.480713  0.913405  0.51274  0.598698 | 22.59012473  22.14369401  23.74221696  23.62746877  20.58796357  169.8363736  23.67143127  23.68322474  20.75811327  22.6629637 |
| IL-5 | 5 | rs11680908  rs6737109  rs72831687  rs73040130  rs7767396 | 2  2  6  19  6 | 110076751  23179531  16092360  36746190  43927050 | G  C  G  C  G | A  T  A  T  A | -0.2634  -0.116  0.5239  -0.2638  -0.1515 | 0.0554  0.0247  0.1109  0.0529  0.0246 | 2.03E-06  2.40E-06  1.69E-06  6.00E-07  7.69E-10 | -0.0661654  -0.0161939  -0.357066  -0.0239591  -0.0287409 | 0.066644773  0.032759746  0.193114399  0.067657385  0.032484601 | 0.320804  0.621078  0.0644597  0.723246  0.376289 | 22.6053904  22.05576226  22.31689163  24.86784996  37.92757287 |
| IL-4 | 9 | rs10512267  rs116705532  rs117146485  rs17713451  rs73023729  rs7613691  rs79597994  rs9508291  rs9941733 | 9  1  9  7  6  3  1  13  20 | 102190129  113705169  138824257  151162472  159654030  147653591  151803047  29710620  374061 | C  G  C  G  G  G  C  C  G | T  T  T  A  A  A  T  T  A | 0.0824  0.4678  0.2924  -0.1274  0.1796  -0.1775  0.5831  0.1676  -0.114 | 0.0161  0.0978  0.0629  0.0253  0.0366  0.0384  0.127  0.0359  0.0229 | 2.94E-07  1.76E-06  2.71E-06  4.97E-07  9.03E-07  4.05E-06  4.32E-06  3.03E-06  6.88E-07 | -0.0235718  -0.0801767  -0.121781  0.0311986  -0.218906  0.0195182  -0.00744803  0.000239572  0.040339 | 0.034728309  0.13187138  0.161096927  0.047301556  0.129297493  0.066604266  0.110403518  0.065127894  0.043479085 | 0.497297  0.543193  0.44968  0.509531  0.0904476  0.769486  0.946214  0.997065  0.353522 | 26.19405116  22.8792996  21.60993426  25.35699667  24.07966795  21.36654324  21.08038998  21.79511332  24.78213611 |
| IL2rα | 10 | rs112407568  rs11241559  rs115360066  rs117244812  rs12722497  rs12722557  rs185231391  rs2182409  rs4733117  rs61705228 | 10  5  5  17  10  10  3  10  8  4 | 6122195  119976700  9823768  6443310  6095928  6070408  59359679  6209218  32137610  101196302 | G  G  G  G  C  C  C  C  C  C | A  T  A  A  A  T  T  T  A  T | 0.2624  0.1264  -0.1867  0.7064  -0.6279  0.3673  -0.8503  -0.12  -0.1369  -0.3303 | 0.05  0.0266  0.0379  0.1488  0.0485  0.0732  0.1809  0.0251  0.0292  0.0716 | 1.46E-07  2.00E-06  8.06E-07  2.10E-06  1.57E-38  5.17E-07  1.47E-06  1.66E-06  2.63E-06  3.99E-06 | 0.0639097  0.0158971  0.0804304  -0.0471307  -0.00495155  0.0240604  0.05568  -0.0259361  0.0026701  0.0066956 | 0.066386293  0.038289892  0.055977494  0.158444981  0.059077558  0.085751749  0.153237601  0.036030407  0.046700694  0.072020496 | 0.335701  0.678012  0.150765  0.766117  0.933204  0.779031  0.716338  0.471624  0.954406  0.925929 | 27.541504  22.58036068  24.26667177  22.53696959  167.6090594  25.17791103  22.09363752  22.85678005  21.98068352  21.28095448 |
| IL-2 | 9 | rs12051139  rs13412535  rs170117  rs2807544  rs4634519  rs61335305  rs62124990  rs7615304  rs80336398 | 16  2  4  1  7  15  2  3  3 | 86918674  224874874  55390380  15204245  67192928  66453074  19238636  156675703  64060934 | C  G  C  G  G  C  G  G  C | T  A  T  A  A  A  T  A  T | 0.1131  -0.1764  0.1617  -0.1175  0.1261  -0.4514  0.6961  0.1172  -0.4001 | 0.0247  0.0332  0.0349  0.0253  0.0269  0.0918  0.1495  0.0242  0.0858 | 4.76E-06  1.18E-07  3.87E-06  3.41E-06  2.77E-06  7.32E-07  3.22E-06  1.21E-06  2.82E-06 | 0.00147553  0.107429  -0.00360519  -0.00798401  -0.0387673  -0.140418  0.259752  -0.011295  0.140014 | 0.033372629  0.03853527  0.048504288  0.033437368  0.035937826  0.121682933  0.105196092  0.03380428  0.151419692 | 0.964734  0.0053066  0.94075  0.81128  0.280707  0.248514  0.0135409  0.738282  0.355135 | 20.966759  28.23065757  21.46689272  21.56923245  21.97483451  24.17896725  21.68008009  23.45440885  21.74515597 |
| IFN-γ | 9 | rs10487554  rs112783231  rs115729819  rs11843756  rs12420286  rs1867282  rs2073438  rs74148555  rs78296352 | 7  8  4  13  11  9  17  10  1 | 149367686  68123274  169704667  49254892  103777894  102172147  6900076  92079842  22821844 | G  G  G  G  C  C  G  C  G | A  A  A  T  T  T  A  T  T | 0.0895  0.2408  -0.2484  -0.184  -0.2376  -0.0774  -0.0898  0.3732  -0.343 | 0.0183  0.0511  0.0515  0.0393  0.0501  0.0166  0.0188  0.0774  0.0652 | 1.09E-06  1.96E-06  1.38E-06  3.09E-06  2.08E-06  3.15E-06  1.68E-06  2.64E-06  1.38E-07 | -0.03824  -0.145338  -0.0361224  0.0531739  0.0612231  0.00486439  0.0492401  -0.0729716  -0.089286 | 0.036152102  0.120638559  0.135484851  0.103105003  0.079995501  0.034976776  0.03572174  0.094602967  0.083674641 | 0.290168  0.228304  0.789765  0.606046  0.444074  0.889391  0.168069  0.440502  0.285944 | 23.91904805  22.20604241  23.26423226  21.9205045  22.49144824  21.74031064  22.81586691  23.24884322  27.67534533 |
| GROα | 10 | rs1113500  rs118158560  rs12075  rs140734053  rs185768063  rs188345231  rs2422841  rs508977  rs62024303  rs78653452 | 1  7  1  10  6  8  20  4  15  20 | 108595442  38146610  159175354  5401459  16494983  41437350  3080352  74762383  88871162  9762055 | G  G  G  G  G  C  G  G  G  G | T  A  A  A  A  T  A  T  A  T | -0.1174  -0.2703  -0.3751  -0.7257  -0.3998  -0.623  0.1657  0.3802  0.3053  0.7362 | 0.0244  0.0594  0.0237  0.1561  0.076  0.1323  0.0361  0.028  0.0666  0.1558 | 1.57E-06  3.42E-06  1.24E-55  3.58E-06  1.46E-07  4.34E-06  4.66E-06  7.56E-42  4.41E-06  1.21E-06 | 0.0136802  -0.0418698  -0.0585035  -0.112112  0.0509616  0.0105534  0.025528  -0.0173469  -0.137965  -0.212128 | 0.033811943  0.069892345  0.032794475  0.124650522  0.183235804  0.121612054  0.052920003  0.03816338  0.079888204  0.147677943 | 0.685774  0.549132  0.0744328  0.368434  0.78092  0.930847  0.62953  0.649438  0.084172  0.150882 | 23.15029562  20.70709621  250.4940626  21.61267088  27.67313712  22.17463117  21.06835429  184.377602  21.01382689  22.32835454 |
| GCSF | 8 | rs115256310  rs11903143  rs147128865  rs1817411  rs2671444  rs74148555  rs76287671  rs77318030 | 5  2  9  8  12  10  19  19 | 71399691  29592460  34972766  98598328  101552075  92079842  44122296  55055897 | G  G  C  C  G  C  C  C | A  A  T  T  A  T  T  T | 0.6821  -0.087  -0.27  -0.089  0.0784  0.3715  -0.0938  0.2045 | 0.136  0.0176  0.0587  0.0191  0.0166  0.0755  0.0189  0.0428 | 6.73E-07  6.35E-07  4.92E-06  3.10E-06  2.48E-06  1.55E-06  6.92E-07  2.21E-06 | 0.0820896  0.0260939  -0.136927  0.0228061  -0.0462919  -0.0729716  -0.0582333  0.0144756 | 0.129936467  0.036381891  0.142098388  0.036945841  0.033713244  0.094602967  0.042011506  0.07491193 | 0.527539  0.473237  0.335243  0.537047  0.169719  0.440502  0.165708  0.846775 | 25.15465019  24.43504649  21.15686553  21.71267235  22.30570475  24.21161353  24.63100137  22.82964124 |
| bFGF | 5 | rs13412535  rs145577605  rs747334  rs75168112  rs9907295 | 2  6  10  18  17 | 224874874  27610011  92744744  71086067  34257313 | G  G  G  C  C | A  A  A  T  T | 0.1112  -0.2081  -0.0751  0.1001  0.1319 | 0.0225  0.0428  0.0164  0.0214  0.0269 | 7.35E-07  9.64E-07  4.53E-06  3.00E-06  7.95E-07 | 0.107429  0.0136058  0.0425965  -0.0586377  -0.0177387 | 0.03853527  0.214085219  0.0325642  0.050729894  0.055650341 | 0.0053066  0.949326  0.190847  0.24773  0.749914 | 24.42556049  23.64049808  20.9696981  21.87966198  24.0427993 |
| Eotaxin | 18 | rs11719635  rs12075  rs1476670  rs2024050  rs2210755  rs2211994  rs2228467  rs2419841  rs3091309  rs342511  rs3960554  rs5746492  rs5754733  rs59808887  rs75426604  rs79722574  rs80341932  rs9317045 | 3  1  1  7  9  21  3  10  3  3  7  22  22  5  14  17  18  13 | 43181148  159175354  44508195  75460393  80223823  18047593  42906116  115335983  46303184  42578509  75845597  18393933  34269594  31846520  35857714  32619052  56915928  59630038 | C  G  C  G  C  C  C  C  G  G  G  G  C  C  C  C  G  C | T  A  A  A  T  T  T  T  A  A  T  A  A  T  A  T  A  A | -0.1307  -0.1671  0.1007  -0.1728  0.1104  -0.0885  0.4163  0.1277  -0.1283  -0.0927  -0.0798  -0.0954  0.1042  0.1673  0.1366  0.1113  -0.1016  -0.1182 | 0.0243  0.0156  0.0217  0.0303  0.0242  0.0177  0.0292  0.0279  0.0203  0.0157  0.0169  0.0207  0.0214  0.0358  0.0291  0.0228  0.0205  0.0237 | 6.16E-08  1.33E-26  3.51E-06  1.10E-08  4.85E-06  6.08E-07  2.27E-46  4.98E-06  3.63E-10  3.60E-09  2.23E-06  3.96E-06  1.06E-06  2.91E-06  2.53E-06  1.06E-06  6.69E-07  5.82E-07 | 0.0287614  -0.0585035  0.00636118  0.034265  0.0983586  -0.0243768  -0.00415055  -0.00131677  -0.00474769  -0.0240252  0.0563257  -0.0417356  -0.0213716  -0.0720366  -0.0167832  -0.0559784  0.00838599  0.0347964 | 0.048129282  0.032794475  0.040540054  0.052911922  0.06525216  0.036796311  0.067422521  0.053565401  0.041379536  0.032378015  0.044803066  0.042503875  0.039148911  0.062422319  0.048061459  0.045035129  0.036028529  0.044563302 | 0.550116  0.0744328  0.875315  0.517253  0.131718  0.507664  0.950913  0.980388  0.908655  0.458074  0.208687  0.326137  0.585131  0.248492  0.726937  0.21387  0.815948  0.434902 | 28.92934681  114.7370562  21.5347321  32.52387021  20.81169319  25  203.2577524  20.94948677  39.94489068  34.86263134  22.29627814  21.24007561  23.70870818  21.83865204  22.03512004  23.82981302  24.56290303  24.87357795 |

SNP, single nucleotide polymorphism; Beta, beta coefficient; SE, standard error; CTACK, cutaneous T-cell attracting chemokine; GROa, Growth-regulated protein alpha; HGF, Hepatocyte growth factor; IP10, Interferon gamma-induced protein 10; IL1ra, Interleukin-1-receptor antagonist; IL-12p70, Interleukin-12p70; IL-16, Interleukin-16; IL-18, Interleukin-18; MIP1b, Macrophage inflammatory protein 1b; MCP1, Monocyte chemoattractant protein-1; PDGFbb, Platelet-derived growth factor BB; RANTES, regulated on Activation, Normal T Cell Expressed and Secreted; SCF, Stem cell factor; SCGFb, Stem cell growth factor beta; TRAIL, TNF-related apoptosis-inducing ligand; TNFb, Tumor necrosis factor beta; VEGF, Vascular endothelial growth factor.

**Supplementary Table S2. SNPs used as instrument variables for endometriosis.**

| **Chr** | **Position** | **SNP** | **Effect Allele** | **Other Allele** | **Beta** | **SE** | **P value** | **F statistics** |
| --- | --- | --- | --- | --- | --- | --- | --- | --- |
| 9  10  5  8  14  2  1  2  12  1  5  19  10  21  9  21  2  5  6 | 22177527  117759669  62671459  40272394  34477410  129048312  214532811  51436672  2092060  82963120  153991994  2421683  19600820  27421510  14798628  36272365  205283241  148690612  49956076 | rs1095899  rs11197495  rs116329098  rs17626927  rs1769574  rs28595286  rs3013455  rs34964547  rs4765871  rs57590076  rs60329486  rs7245759  rs72782359  rs73183033  rs74869083  rs76621869  rs78517472  rs920318  rs9369929 | T  C  A  G  T  A  T  A  T  C  T  T  C  A  C  C  C  T  C | G  A  C  A  G  G  C  G  C  T  C  G  T  G  T  T  A  C  T | 0.163245  0.323541  0.458497  0.224138  -0.175745  0.263664  -0.223877  0.258252  0.163348  0.172181  0.17821  0.198545  0.608964  -0.190754  0.396747  0.372637  0.656091  -0.290091  0.162836 | 0.0349802  0.0681181  0.0945413  0.0442738  0.0383461  0.0560394  0.0404714  0.0558848  0.0353537  0.0369119  0.0346032  0.0414104  0.125148  0.0414468  0.0796338  0.0789725  0.14033  0.0627081  0.0348839 | 3.06E-06  2.04E-06  1.24E-06  4.14E-07  4.58E-06  2.54E-06  3.17E-08  3.82E-06  3.83E-06  3.09E-06  2.60E-07  1.63E-06  1.14E-06  4.18E-06  6.29E-07  2.38E-06  2.93E-06  3.73E-06  3.04E-06 | 21.77886285  22.5597143  23.51959598  25.62933791  21.00504204  22.13679378  30.60007574  21.35502013  21.34803461  21.75893578  26.52354777  22.98788724  23.67747635  21.18191868  24.82175147  22.26487066  21.85883995  21.40036722  21.78967665 |

Chr, chromosome; SNP, single nucleotide polymorphism; EAF, effect allele frequency; SE, standard error

**Supplementary Table S3. MR analysis of systemic inflammatory regulators and endometriosis risk**

| **systematic inflammatory regulators** | | **Number of SNPs** | **OR (95% CI)** | **P** | **P for heterogeneity test** | **P for MR-Egger intercept** |
| --- | --- | --- | --- | --- | --- | --- |
| CTACK  βNGF  VEGF  MIF  TRAIL  TNFβ  TNFα  SDF1α  SCGFβ  SCF  IL-16  RANTES  PDGFbb  MIP1β  MIP1α  MIG  MCSF  MCP3  MCP1  IL-12p70  IP10  IL-18  IL-17  IL-13  IL-10  IL-8  IL-6  IL1ra  IL-1β  HGF  IL-9  IL-7  IL-5  IL-4  IL2rα  IL-2  IFN-γ  GROα  GCSF  bFGF  Eotaxin | Inverse variance weighted  MR Egger  Weighted median  Inverse variance weighted  MR Egger  Weighted median  Inverse variance weighted  MR Egger  Weighted median  Inverse variance weighted  MR Egger  Weighted median  Inverse variance weighted  MR Egger  Weighted median  Inverse variance weighted  MR Egger  Weighted median  Inverse variance weighted  MR Egger  Weighted median  Inverse variance weighted  MR Egger  Weighted median  Inverse variance weighted  MR Egger  Weighted median  Inverse variance weighted  MR Egger  Weighted median  Inverse variance weighted  MR Egger  Weighted median  Inverse variance weighted  MR Egger  Weighted median  Inverse variance weighted  MR Egger  Weighted median  Inverse variance weighted  MR Egger  Weighted median  Inverse variance weighted  MR Egger  Weighted median  Inverse variance weighted  MR Egger  Weighted median  Inverse variance weighted  MR Egger  Weighted median  Inverse variance weighted  MR Egger  Weighted median  Inverse variance weighted  MR Egger  Weighted median  Inverse variance weighted  MR Egger  Weighted median  Inverse variance weighted  MR Egger  Weighted median  Inverse variance weighted  MR Egger  Weighted median  Inverse variance weighted  MR Egger  Weighted median  Inverse variance weighted  MR Egger  Weighted median  Inverse variance weighted  MR Egger  Weighted median  Inverse variance weighted  MR Egger  Weighted median  Inverse variance weighted  MR Egger  Weighted median  Inverse variance weighted  MR Egger  Weighted median  Inverse variance weighted  MR Egger  Weighted median  Inverse variance weighted  MR Egger  Weighted median  Inverse variance weighted  MR Egger  Weighted median  Inverse variance weighted  MR Egger  Weighted median  Inverse variance weighted  MR Egger  Weighted median  Inverse variance weighted  MR Egger  Weighted median  Inverse variance weighted  MR Egger  Weighted median  Inverse variance weighted  MR Egger  Weighted median  Inverse variance weighted  MR Egger  Weighted median  Inverse variance weighted  MR Egger  Weighted median  Inverse variance weighted  MR Egger  Weighted median  Inverse variance weighted  MR Egger  Weighted median  Inverse variance weighted  MR Egger  Weighted median | 12  12  12  7  7  7  19  19  19  6  6  6  26  26  26  6  6  6  5  5  5  8  8  8  16  16  16  9  9  9  10  10  10  10  10  10  18  18  18  44  44  44  8  8  8  14  14  14  8  8  8  3  3  3  18  18  18  11  11  11  12  12  12  17  17  17  10  10  10  12  12  12  11  11  11  4  4  4  4  4  4  6  6  6  6  6  6  7  7  7  6  6  6  10  10  10  5  5  5  9  9  9  10  10  10  9  9  9  9  9  9  10  10  10  8  8  8  5  5  5  18  18  18 | 1.118(0.979,1.276)  1.056(0.806,1.383)  1.118(0.931,1.342)  0.885(0.694,1.130)  2.188(0.745,6.417)  0.910(0.696,1.190)  0.984(0.889,1.090)  1.114(0.929,1.336)  1.039(0.918,1.176)  0.979(0.792,1.209)  1.039(0.737,1.465)  0.949(0.726,1.241)  0.993(0.902,1.094)  0.943(0.831,1.071)  0.963(0.858,1.080)  1.022(0.921,1.133)  1.047(0.899,1.221)  1.032(0.912,1.166)  1.014(0.831,1.236)  0.971(0.682,1.383)  0.955(0.748,1.221)  0.849(0.634,1.138)  1.055(0.594,1.874)  0.900(0.613,1.320)  1.119(0.998,1.255)  1.116(0.908,1.372)  1.106(0.946,1.294)  1.085(0.742,1.589)  1.342(0.569,3.165)  0.992(0.694,1.420)  1.043(0.936,1.162)  1.019(0.852,1.219)  1.016(0.887,1.163)  1.000(0.846,1.181)  0.757(0.476,1.206)  0.931(0.741,1.171)  0.856(0.742,0.987)  0.711(0.543,0.931)  0.760(0.628,0.919)  0.977(0.908,1.050)  1.025(0.911,1.154)  0.965(0.860,1.082)  1.048(0.857,1.280)  1.164(0.671,2.020)  1.029(0.798,1.328)  0.921(0.790,1.075)  0.851(0.614,1.181)  0.971(0.800,1.179)  1.112(0.980,1.262)  1.141(0.907,1.436)  1.146(0.975,1.346)  1.029(0.780,1.359)  1.605(0.541,4.764)  1.017(0.787,1.315)  1.073(0.926,1.245)  1.167(0.848,1.605)  1.092(0.892,1.335)  1.120(0.976,1.284)  1.102(0.878,1.383)  1.087(0.929,1.271)  1.009(0.868,1.174)  1.159(0.855,1.571)  0.980(0.799,1.201)  1.002(0.905,1.111)  1.064(0.875,1.294)  1.006(0.866,1.168)  0.936(0.722,1.215)  1.130(0.666,1.919)  0.876(0.637,1.204)  1.022(0.918,1.138)  0.945(0.782,1.141)  1.047(0.911,1.202)  1.009(0.855,1.190)  1.282(0.905,1.817)  1.095(0.889,1.350)  1.193(0.905,1.571)  0.873(0.621,1.229)  1.142(0.892,1.461)  1.351(1.015,1.797)  1.118(0.789,1.767)  1.267(0.931,1.724)  1.138(0.918,1.412)  1.508(0.832,2.735)  1.103(0.841,1.448)  1.045(0.816,1.338)  1.588(0.930,2.710)  1.090(0.802,1.483)  0.929(0.733,1.178)  0.627(0.360,1.092)  0.868(0.644,1.171)  0.904(0.733,1.115)  0.991(0.587,1.670)  0.932(0.710,1.224)  1.071(0.944,1.215)  1.158(0.831,1.614)  1.091(0.927,1.284)  1.089(0.841,1.409)  0.786(0.394,1.567)  1.175(0.877,1.576)  0.850(0.682,1.060)  0.965(0.662,1.408)  0.890(0.665,1.191)  1.004(0.889,1.133)  0.969(0.794,1.183)  1.000(0.856,1.166)  1.008(0.793,1.283)  1.381(0.921,2.071)  1.017(0.778,1.330)  0.863(0.682,1.093)  1.055(0.691,1.610)  0.816(0.587,1.137)  1.024(0.920,1.139)  1.032(0.799,1.331)  1.161(1.009,1.335)  0.991(0.791,1.241)  1.126(0.795,1.597)  1.079(0.794,1.468)  1.083(0.563,2.085)  3.597(0.176,73.479)  0.828(0.454,1.508)  1.003(0.864,1.165)  1.008(0.688,1.477)  0.990(0.796,1.231) | 0.099904551  0.703139771  0.230914804  0.326119932  0.213368132  0.4909498  0.757482703  0.260984738  0.549105924  0.843488048  0.837453555  0.702718991  0.888054667  0.376304825  0.520094724  0.682259652  0.584626331  0.619860487  0.89351536  0.880716357  0.715765364  0.273602748  0.861646647  0.589930617  0.054021979  0.314865841  0.204945235  0.674084762  0.522400914  0.96625217  0.446341244  0.837764122  0.817733668  0.997737245  0.27541486  0.542720996  0.032361532  0.024542196  0.004849692  0.521015096  0.681794517  0.541854859  0.649146322  0.607820206  0.824290781  0.297806305  0.353535923  0.766640358  0.099862536  0.304292457  0.097269497  0.838977536  0.550587249  0.895776193  0.348551092  0.357026445  0.3936126  0.10535416  0.423174173  0.300680664  0.910019219  0.364796362  0.842860082  0.967978606  0.541132418  0.94157389  0.620446025  0.662677266  0.415418358  0.690955477  0.569929855  0.517604637  0.91946266  0.195633599  0.394940563  0.21031702  0.517505965  0.292652476  0.039082031  0.503295424  0.132060102  0.239879432  0.247354028  0.478093247  0.728127809  0.165618129  0.582642091  0.546430814  0.159884256  0.354850688  0.347411206  0.974492362  0.614277856  0.286312548  0.411800417  0.295066066  0.518507054  0.543315091  0.279900296  0.149426564  0.85936668  0.43287685  0.953681672  0.763141806  0.999512584  0.945486456  0.162235609  0.902366833  0.221051363  0.811665048  0.229077598  0.665661389  0.817168211  0.137555245  0.940133329  0.52995064  0.626891961  0.810660611  0.466617758  0.536784764  0.966317144  0.968075039  0.928098999 | 0.787408593  0.733393124  0.197809506  0.359190275  0.892592623  0.956103169  0.922564311  0.873224924  0.06009858  0.072871282  0.537839488  0.420298083  0.370013768  0.246462382  0.522948358  0.492335359  0.931785127  0.899849373  0.002581664  0.00191321  0.699578947  0.613653793  0.697839079  0.774030492  0.368583711  0.462496843  0.591481274  0.594446924  0.927538276  0.886822763  0.137953775  0.112661163  0.809590076  0.721406638  0.151791856  0.135772275  0.581653168  0.535216225  0.803801485  0.729291557  0.664895592  0.680079319  0.44916175  0.414168369  0.136781913  0.126689046  0.711544651  0.721256893  0.745991617  0.881070362  0.138114667  0.766659179  0.376736989  0.422087931  0.68861428  0.720573817  0.448521277  0.781410516  0.690584661  0.910061479  0.521601677  0.398972182  0.973430719  0.961683761  0.283978661  0.286408453  0.893183838  0.891960338  0.911635267  0.874295135  0.026338658  0.102678436  0.68653605  0.733799316  0.360372786  0.27399734  0.465492074  0.447938152  0.029272811  0.031014051  0.683573206  0.616173557 | 0.643852499  0.1534776  0.125466224  0.688138758  0.23891359  0.689217241  0.776833622  0.424402898  0.973327765  0.599820114  0.763078339  0.245649371  0.136217778  0.313282084  0.700768317  0.597971904  0.803424416  0.557720092  0.570159916  0.868072751  0.327622714  0.486235879  0.443862356  0.346646414  0.159435453  0.155394635  0.452494414  0.375800869  0.158938131  0.184208973  0.725888377  0.632094914  0.391884742  0.445781575  0.672962899  0.116982071  0.299112708  0.949911569  0.386030132  0.481881942  0.979407388 |
|  |  |  |  |  |  |  |

Abbreviations: SNP, single nucleotide polymorphism; OR, odds ratio; CI, confidence interval.

**Supplementary Table S4. MR analysis of the association between endometriosis and systemic inflammatory regulators**

| **systematic inflammatory regulators** | | | **Number of SNPs** | **β (95% CI)** | **P** | **P for heterogeneity test** | **P for MR-Egger intercept** |
| --- | --- | --- | --- | --- | --- | --- | --- |
| CTACK  βNGF  VEGF  MIF  TRAIL  TNFβ  TNFα  SDF1α  SCGFβ  SCF  IL-16  RANTES  PDGFbb  MIP1β  MIP1α  MIG  MCSF  MCP3  MCP1  IL-12p70  IP10  IL-18  IL-17  IL-13  IL-10  IL-8  IL-6  IL1ra  IL-1β  HGF  IL-9  IL-7  IL-5  IL-4  IL2rα  IL-2  IFN-γ  GROα  GCSF  bFGF  Eotaxin | | Inverse variance weighted  MR Egger  Weighted median  Inverse variance weighted  MR Egger  Weighted median  Inverse variance weighted  MR Egger  Weighted median  Inverse variance weighted  MR Egger  Weighted median  Inverse variance weighted  MR Egger  Weighted median  Inverse variance weighted  MR Egger  Weighted median  Inverse variance weighted  MR Egger  Weighted median  Inverse variance weighted  MR Egger  Weighted median  Inverse variance weighted  MR Egger  Weighted median  Inverse variance weighted  MR Egger  Weighted median  Inverse variance weighted  MR Egger  Weighted median  Inverse variance weighted  MR Egger  Weighted median  Inverse variance weighted  MR Egger  Weighted median  Inverse variance weighted  MR Egger  Weighted median  Inverse variance weighted  MR Egger  Weighted median  Inverse variance weighted  MR Egger  Weighted median  Inverse variance weighted  MR Egger  Weighted median  Inverse variance weighted  MR Egger  Weighted median  Inverse variance weighted  MR Egger  Weighted median  Inverse variance weighted  MR Egger  Weighted median  Inverse variance weighted  MR Egger  Weighted median  Inverse variance weighted  MR Egger  Weighted median  Inverse variance weighted  MR Egger  Weighted median  Inverse variance weighted  MR Egger  Weighted median  Inverse variance weighted  MR Egger  Weighted median  Inverse variance weighted  MR Egger  Weighted median  Inverse variance weighted  MR Egger  Weighted median  Inverse variance weighted  MR Egger  Weighted median  Inverse variance weighted  MR Egger  Weighted median  Inverse variance weighted  MR Egger  Weighted median  Inverse variance weighted  MR Egger  Weighted median  Inverse variance weighted  MR Egger  Weighted median  Inverse variance weighted  MR Egger  Weighted median  Inverse variance weighted  MR Egger  Weighted median  Inverse variance weighted  MR Egger  Weighted median  Inverse variance weighted  MR Egger  Weighted median  Inverse variance weighted  MR Egger  Weighted median  Inverse variance weighted  MR Egger  Weighted median  Inverse variance weighted  MR Egger  Weighted median  Inverse variance weighted  MR Egger  Weighted median  Inverse variance weighted  MR Egger  Weighted median | 18  18  18  18  18  18  18  18  18  18  18  18  18  18  18  15  15  15  18  18  18  18  18  18  17  17  17  18  18  18  18  18  18  18  18  18  18  18  18  18  18  18  18  18  18  18  18  18  18  18  18  16  16  16  18  18  18  18  18  18  18  18  18  18  18  18  18  18  18  18  18  18  18  18  18  18  18  18  18  18  18  18  18  18  19  19  19  18  18  18  18  18  18  18  18  18  18  18  18  18  18  18  18  18  18  18  18  18  18  18  18  18  18  18  18  18  18  18  18  18  17  17  17 | 0.038(-0.048,0.125)  0.007(-0.291,0.304)  0.031(-0.073,0.135)  0.080(-0.012,0.171)  -0.086(-0.409,0.237)  0.058(-0.049,0.165)  0.009(-0.046,0.065)  0.091(-0.102,0.283)  -0.004(-0.075,0.068)  -0.058(-0.137,0.020)  -0.348(-0.595,-0.102)  -0.056(-0.160,0.049)  -0.027(-0.077,0.023)  -0.170(-0.333,-0.008)  -0.047(-0.114,0.020)  -0.042(-0.188,0.103)  0.164(-0.460,0.788)  -0.060(-0.233,0.113)  0.005(-0.066,0.077)  0.066(-0.148,0.280)  0.003(-0.095,0.100)  0.007(-0.034,0.049)  -0.007(-0.165,0.152)  0.014(-0.046,0.074)  0.036(-0.044,0.117)  0.183(-0.121,0.487)  0.017(-0.087,0.122)  -0.002(-0.048,0.045)  -0.071(-0.230,0.088)  0.009(-0.051,0.069)  -0.005(-0.083,0.074)  -0.085(-0.357,0.186)  -0.007(-0.115,0.100)  0.022(-0.056,0.099)  0.206(-0.084,0.495)  0.016(-0.092,0.125)  0.017(-0.021,0.055)  0.008(-0.132,0.148)  0.014(-0.042,0.069)  0.022(-0.035,0.080)  0.013(-0.196,0.222)  0.031(-0.037,0.100)  0.025(-0.050,0.099)  0.138(-0.117,0.393)  0.015(-0.088,0.119)  0.035(-0.036,0.106)  -0.051(-0.301,0.199)  0.023(-0.069,0.114)  0.053(-0.058,0.164)  0.124(-0.283,0.531)  -0.013(-0.157,0.131)  -0.001(-0.164,0.163)  -0.488(-1.078,0.101)  -0.015(-0.209,0.179)  -0.018(-0.066,0.031)  -0.022(-0.193,0.150)  -0.032(-0.096,0.033)  0.012(-0.040,0.064)  0.013(-0.177,0.203)  0.030(-0.040,0.100)  0.028(-0.044,0.100)  -0.110(-0.354,0.134)  0.031(-0.073,0.136)  0.029(-0.043,0.100)  0.055(-0.189,0.300)  0.036(-0.056,0.127)  0.021(-0.027,0.069)  0.027(-0.136,0.191)  0.016(-0.052,0.084)  0.026(-0.049,0.102)  0.087(-0.151,0.326)  -0.003(-0.104,0.098)  0.017(-0.034,0.069)  0.117(-0.057,0.290)  0.027(-0.043,0.097)  0.035(-0.045,0.115)  0.080(-0.196,0.357)  0.026(-0.080,0.132)  0.001(-0.047,0.050)  -0.058(-0.215,0.100)  0.009(-0.050,0.069)  -0.011(-0.068,0.045)  0.033(-0.090,0.156)  -0.002(-0.083,0.078)  0.005(-0.018,0.029)  0.030(-0.019,0.079)  0.012(-0.018,0.041)  -0.012(-0.060,0.037)  -0.072(-0.235,0.092)  -0.027(-0.093,0.038)  0.004(-0.036,0.044)  0.111(-0.123,0.344)  0.001(-0.050,0.052)  0.022(-0.054,0.099)  0.084(-0.191,0.358)  0.008(-0.104,0.120)  0.015(-0.054,0.084)  -0.097(-0.339,0.146)  0.005(-0.098,0.108)  0.021(-0.030,0.072)  0.037(-0.136,0.210)  0.022(-0.043,0.087)  0.003(-0.070,0.076)  -0.044(-0.293,0.206)  0.044(-0.062,0.150)  0.024(-0.047,0.095)  0.165(-0.073,0.402)  0.049(-0.046,0.144)  0.037(-0.019,0.092)  0.064(-0.135,0.263)  0.056(-0.020,0.132)  -0.000(-0.075,0.075)  -0.056(-0.308,0.195)  0.021(-0.081,0.122)  -0.003(-0.057,0.052)  -0.058(-0.246,0.130)  0.035(-0.038,0.108)  0.013(-0.042,0.068)  -0.001(-0.195,0.193)  0.020(-0.051,0.092)  0.015(-0.036,0.066)  -0.072(-0.241,0.096)  0.016(-0.054,0.085) | 0.388033487  0.96640218  0.560055902  0.086964478  0.608246594  0.29010612  0.743806043  0.369674412  0.923383503  0.14440848  0.01360829  0.295788982  0.288381257  0.056369277  0.166468776  0.568891742  0.615143916  0.499741066  0.887175191  0.553011272  0.955859816  0.725811759  0.936645519  0.645090572  0.376866692  0.256470215  0.745247665  0.943891569  0.396751398  0.761372173  0.91042995  0.546807967  0.893937073  0.585035626  0.183004162  0.768101016  0.378140698  0.911569412  0.626036344  0.446924518  0.905810558  0.373380271  0.517297441  0.305351838  0.769613081  0.33421861  0.696059292  0.630847084  0.349826944  0.558029593  0.859404163  0.994190958  0.12683217  0.87917529  0.479680083  0.807502696  0.336172067  0.650755343  0.898312177  0.402448351  0.448232307  0.389051371  0.556781567  0.428455593  0.663318935  0.443082689  0.38930259  0.746207045  0.642263799  0.496158276  0.484047105  0.952274022  0.511431882  0.206521194  0.442918186  0.394961523  0.57623612  0.633284191  0.961846527  0.481980171  0.761400919  0.694967203  0.609079841  0.956946994  0.661699848  0.24103844  0.439161426  0.633151923  0.405355512  0.412497016  0.839589434  0.366868301  0.983000639  0.566999575  0.559760137  0.887211803  0.67139716  0.446491613  0.923093793  0.41447193  0.679751034  0.502932384  0.934584083  0.734692389  0.414302798  0.507920677  0.19279448  0.314520423  0.1960806  0.536759335  0.145453175  0.994931069  0.665977916  0.685981255  0.925268383  0.552067606  0.34228615  0.637611488  0.992762664  0.574687771  0.567552122  0.414150078  0.657529891 | 0.08020289  0.059635106  0.104863486  0.114066531  0.230983629  0.222032886  0.305396043  0.630827933  0.357311254  0.50797914  0.097796376  0.084484433  0.300591485  0.26458363  0.909604169  0.875740177  0.255528997  0.25756093  0.934812592  0.936804049  0.247588681  0.21699891  0.518890795  0.569062945  0.854801446  0.808466974  0.070213142  0.050947566  0.588362461  0.578522646  0.948728878  0.941933108  0.085549225  0.065944595  0.10636746  0.191084695  0.875268897  0.832027579  0.248512611  0.197720519  0.367042938  0.39060944  0.996727212  0.994258169  0.709727787  0.644539901  0.289851811  0.250405157  0.393847652  0.418532846  0.236563491  0.193335459  0.99177144  0.991870608  0.695966754  0.675197032  0.312179201  0.333281227  0.569422508  0.53902848  0.778329507  0.775694966  0.722002348  0.672458847  0.530479084  0.522950197  0.99674545  0.994205255  0.677923162  0.621207456  0.52851437  0.565017254  0.237248211  0.192100103  0.365270376  0.315770668  0.196865594  0.170851364  0.266797261  0.214905576  0.917016231  0.932136444 | 0.830149437  0.310204178  0.399191255  0.027935938  0.088828411  0.516558369  0.560675432  0.860105395  0.342396677  0.387034064  0.550278666  0.214421126  0.896930691  0.926453522  0.376439252  0.49253076  0.725413154  0.115140292  0.961042771  0.99575275  0.26230305  0.827543725  0.937743889  0.603594415  0.257929563  0.739221874  0.451418826  0.441777437  0.268524471  0.465942495  0.377680202  0.655903731  0.361259515  0.852253482  0.704789398  0.241231283  0.781431003  0.651704312  0.552029234  0.884118723  0.305197448 |
|  |  | |  |  |  |  |  |

Abbreviations: SNP, single nucleotide polymorphism; CI, confidence interval.
